# Associative visual learning by tethered bees in a controlled visual environment

**DOI:** 10.1038/s41598-017-12631-w

**Published:** 2017-10-10

**Authors:** Alexis Buatois, Cécile Pichot, Patrick Schultheiss, Jean-Christophe Sandoz, Claudio R. Lazzari, Lars Chittka, Aurore Avarguès-Weber, Martin Giurfa

**Affiliations:** 1 Research Centre on Animal Cognition, Center for Integrative Biology, CNRS, University of Toulouse, 118 route de Narbonne, F-31062 Toulouse cedex 09, France; 2 Laboratory Evolution Genomes Behavior and Ecology, CNRS, Univ Paris-Sud, IRD, University Paris Saclay, F-91198 Gif-sur-Yvette, France; 3 Institut de Recherche sur la Biologie de l’Insecte, UMR 7261 CNRS, University François Rabelais of Tours, F-37200 Tours, France; 4 Queen Mary University of London, School of Biological and Chemical Sciences, Biological and Experimental Psychology, Mile End Road, London, E1 4NS United Kingdom

## Abstract

Free-flying honeybees exhibit remarkable cognitive capacities but the neural underpinnings of these capacities cannot be studied in flying insects. Conversely, immobilized bees are accessible to neurobiological investigation but display poor visual learning. To overcome this limitation, we aimed at establishing a controlled visual environment in which tethered bees walking on a spherical treadmill learn to discriminate visual stimuli video projected in front of them. Freely flying bees trained to walk into a miniature Y-maze displaying these stimuli in a dark environment learned the visual discrimination efficiently when one of them (CS+) was paired with sucrose and the other with quinine solution (CS−). Adapting this discrimination to the treadmill paradigm with a tethered, walking bee was successful as bees exhibited robust discrimination and preferred the CS+ to the CS− after training. As learning was better in the maze, movement freedom, active vision and behavioral context might be important for visual learning. The nature of the punishment associated with the CS− also affects learning as quinine and distilled water enhanced the proportion of learners. Thus, visual learning is amenable to a controlled environment in which tethered bees learn visual stimuli, a result that is important for future neurobiological studies in virtual reality.

## Introduction

Decades of research on associative learning and memory have established a few insect species as standard models for the study of these capacities. Fruit flies, bees, crickets and ants, among others, can learn simple associations and their neural underpinnings can be understood at the cellular and molecular levels^1–7^. Among these, the honeybee *Apis mellifera* is a powerful model for the study of various forms of visual and olfactory learning^2,8–10^. In the olfactory domain, a Pavlovian conditioning protocol^2,11,12^ is useful to dissect associative learning in bees at the circuit and molecular levels^2,8,9,13–15^. In this protocol, individually harnessed hungry bees, which extend their proboscis (proboscis extension response - PER) if sugar water touches their antennae, learn to associate an odor presentation with sugar and thus respond with PER to the odor alone. The sugar water acts as an unconditioned stimulus (US) and the odor as the conditioned stimulus (CS). The fact that bees learn and memorize odors despite being immobilized constitutes the key to success of this protocol as it allows combining behavioral recordings with a variety of invasive techniques to characterize neural activity during olfactory acquisition and retention^8,9^.

In the visual domain, freely flying honey bees can be trained to solve visual problems of high complexity in experimental setups where they are rewarded with sucrose solution for the correct choice of visual stimuli (colors, shapes and patterns, depth and motion cues, among others^8,16–18^). The protocols used reveal that when bees are confronted with complex visual tasks, they show learning capacities on a par with those of some vertebrates^10,19–26^. Indeed, freely flying bees can categorize objects based on common visual features^21,23,27^, and master non-elemental discriminations based on relational concepts such as identity (“sameness” and “difference” relationships)^22^, numerosity^28–30^ or on spatial concepts such as “above” or “below” with respect to a constant reference^19,26,31^. Yet, the exploration of the neural bases of visual cognitive processing is not possible in freely flying insects. Attempts to establish a visual variant of PER conditioning have been disappointing until now, as the resulting learning performances are usually poor, even in simple color discrimination tasks (see review in^32^). It seems, therefore, that full immobilization, as imposed by PER conditioning, restricts the possibility of studying more sophisticated forms of visual learning in a way that would permit neurophysiological recordings. Thus, developing alternative protocols that overcome the historical limitations that have impeded understanding and characterizing the neural architecture underlying visual learning in bees is crucial.

Air-cushioned spheres used as treadmills and virtual environments have become popular to study a series of stereotyped responses to olfactory and visual stimuli in a variety of arthropods^33–43^. Yet, attempts to reproduce associative learning in this context have been so far unsuccessful or limited to the observation of learning that occurred beforehand, in unrestrained conditions^38,40^. Here we introduce an experimental protocol in which a tethered bee walking stationary on a treadmill learns to discriminate an appetitive from an aversive visual stimulus in a controlled visual surrounding. We asked 1) if freely flying bees trained to walk into a miniature Y-maze displaying video projected stimuli in a dark environment learn efficiently the visual discrimination when one of them (CS+) is paired with sucrose and the other with quinine solution (CS−); 2) if the miniature-maze situation is adaptable to the controlled environment provided by the treadmill, i.e. if tethered, walking bees learn associations between visual stimuli and reinforcement/punishment in this paradigm; and 3) if the nature of the punishment associated with the aversive visual stimulus influences learning performances in the treadmill.

We report a systematic analysis of the influence of several experimental factors such as phototactic responses, reinforcement quality and tethered condition vs. free walking. We discuss the necessity of active vision for visual discrimination learning in tethered bees. In active vision, an observer varies his viewpoint in order to investigate the environment and extract better information from it. This strategy is used by flying bees^24,44–47^ to extract the borders of objects for better recognition^48,49^ via a series of flight maneuvers. We thus consider our results from this perspective and discuss the extent to which tethering preparations allow for active vision compared to free-flight conditions. More generally, we identify the appropriate conditions for the bees to successfully learn a visual discrimination under these novel experimental conditions, and highlight the importance of aversive reinforcement and active vision for visual learning in honey bees.

## Results

### Experiment 1: visual discrimination under free walking conditions

**Figure 1:**
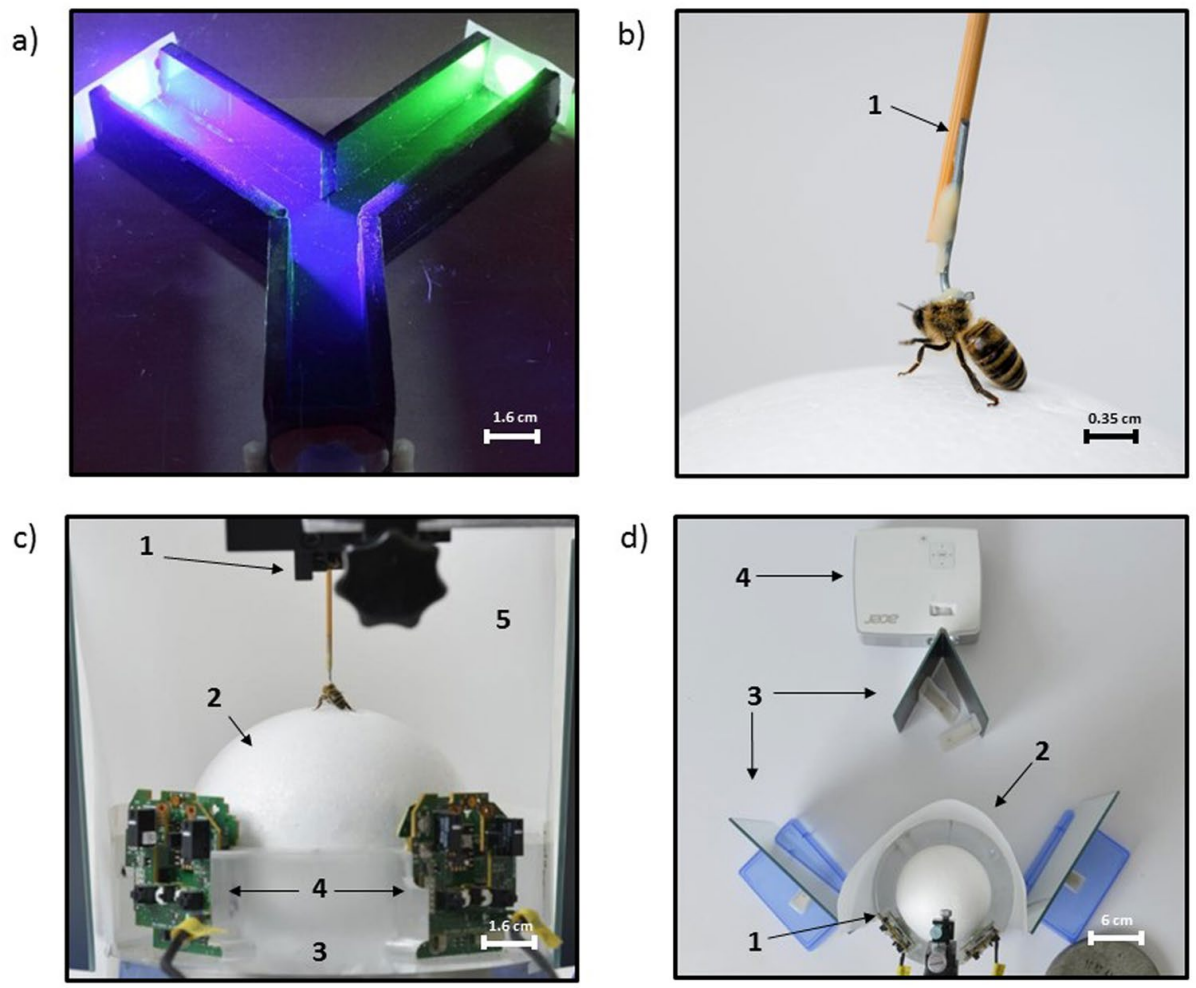
Experimental setups: the mini Y-maze (**a**) and the spherical treadmill (**b**–**d**). (**a**) Top view of the mini Y-maze. Arms were 5 cm in length and 3 × 3 cm in section. Bees walked inside the maze to collect sucrose solution. Stimuli were projected by the video projector onto paper screens placed at the ends of the maze arms. **(b)** A bee tethered by the thorax by means of a vertical attachment (1) made of a wooden toothpick and an L-shaped metal piece glued to the thorax. The toothpick was held by a micromanipulator, which allowed adjusting the position of the bee on the treadmill (© Cyril Frésillon/CNRS). **(c)** The bee held by the micromanipulator (1) walked stationary on the treadmill. The setup was composed of a polystyrene ball (2) floating on a constant airflow (3). Two optic-mouse sensors (4) were placed on the ball support, at 90° of each other to record the ball movements. The setup translates the movements of the walking bee into rotations of the ball (© Cyril Frésillon/CNRS). **(d)** Top view of the treadmill (1) placed behind a semi cylindrical paper screen (2) onto which visual stimuli were projected. A set of mirrors (3) was placed between the video projector (4) and the screen to allow projecting the stimuli on the lateral parts of the semicircular screen without deforming their shapes (© Cyril Frésillon/CNRS).

Efficient and sophisticated learning performances have been observed in freely flying bees in contrast to their poor performance in visual PER conditioning, which imposes total immobility, except for the antennae and mouthparts (see Introduction). The establishment of a controlled treadmill paradigm with a tethered, walking bee discriminating video projected stimuli while being in the darkness requires demonstrating that freely moving bees set in comparable conditions learn to discriminate visual stimuli based on differential reinforcement. We thus investigated to what extent these conditions affect the behavioral performance of freely walking bees trained to collect sucrose solution in a miniature maze (Fig. 1a).

The bees (n = 21) were marked with a color spot on the thorax to facilitate individual recognition upon video recording of behavior. They flew freely between the hive and the laboratory and once they entered the maze set in a dark surrounding, they could only walk due to the maze’s reduced size. The visual stimuli, a blue square (RGB:-1,-1, 1) and a green disc (RGB:-1, 0.2,-1), were projected onto tracing-paper screens placed vertically at the ends of the two arms using a video projector (Acer K135i, Roissy, France). Stimulus intensity was 14000 µW/cm^2^ for the blue square and 1800 µW/cm^2^ for the green disc; these values were adjusted to suppress a homogeneous spontaneous preference for the green disc uncovered by preliminary assays. The blue square and the green disc displayed the same total area (5.5 cm^2^) and subtended a similar visual angle to the bee eyes (square, from edge to edge: 26.3°; disc: 29.6°). This angular range ensures that bees engaged their chromatic vision to achieve the visual discrimination^50,51^. Small Eppendorf tube covers containing 1 M sucrose solution or 60 mM quinine solution were placed at the end of the arm presenting the training visual stimulus. The setup was placed under a red ceiling to ensure a dark environment for the bees. Thus, bees were trained to choose video projected stimuli while walking, similarly to the situation imposed by a treadmill to a tethered bee. Maze bees were, nevertheless, free and returned to the hive between foraging bouts.

**Figure 2:**
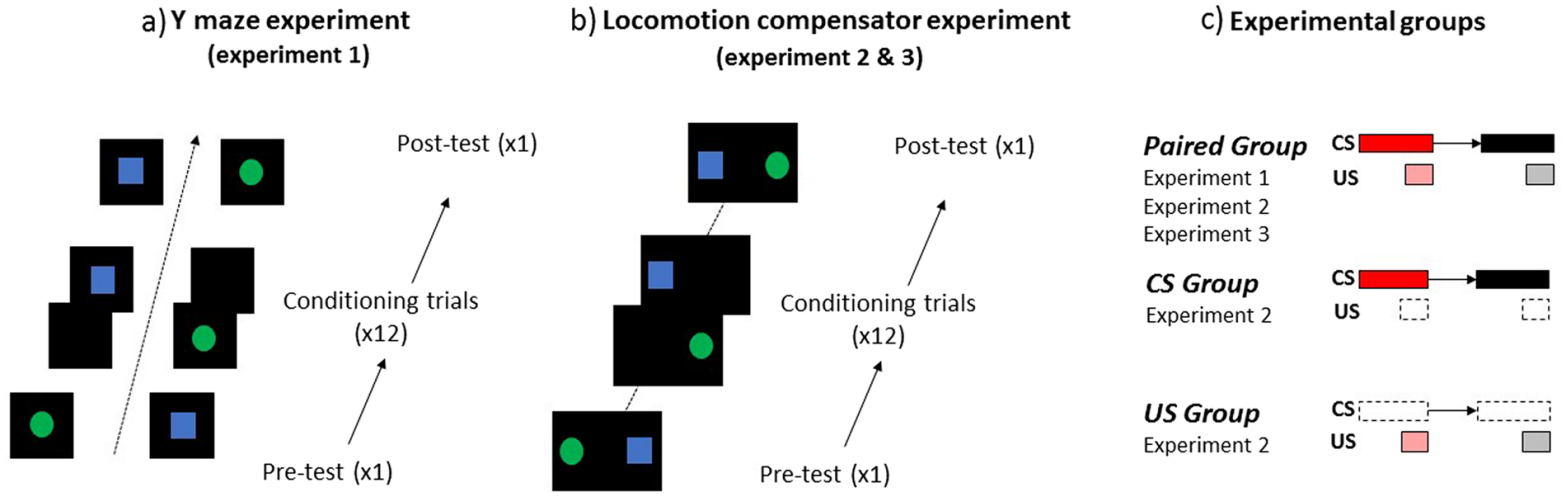
Training, testing and experimental groups at the miniature Y-maze and at the spherical treadmill. **(a)** Experimental sequence at the Y-maze [Experiment 1]. Experiments started with a pre-test in which both stimuli were shown in the absence of reinforcement to check for spontaneous preferences. The pre-test was followed by 12 conditioning trials in which a single reinforced stimulus was shown in one arm of the maze. A black screen was visible in the alternative arm of the maze. After conditioning, a post-test in which both stimuli were shown simultaneously in the absence of reinforcement allowed to determine whether bees learned the visual discrimination. **(b)** Experimental sequence at the treadmill (Experiments 2 and 3). Experiments started with a pre-test in which both stimuli were shown simultaneously in the absence of reinforcement to check for spontaneous preferences. The pre-test was followed by 12 conditioning trials in which a single reinforced stimulus was shown at a time. After conditioning, a post-test in which both stimuli were shown simultaneously in the absence of reinforcement allowed to determine whether bees learned the visual discrimination. **(c)** Experimental groups. In Experiments 1, 2 and 3, the performance of a *paired group* was studied. Bees in this group experienced one visual stimulus (CS+, red rectangle) paired with sucrose solution (pink square) and another visual stimulus (CS−, black rectangle) paired with quinine solution (grey square). In Experiment 2, the performances of a *CS group* and of a *US group* were also studied. Bees in the *CS group* experienced only visual stimulation (CS1, CS2) matching that of the *paired group* but without any reinforcement. Bees in the *US group* experienced a sequence of 12 reinforcements (6 sucrose, and 6 quinine) matching that of the *paired group*.

We first evaluated spontaneous preferences between the blue square and the green disc in a pre-test in which both stimuli were presented simultaneously in the absence of reinforcement (Fig. 2a). Afterwards, we conditioned bees to discriminate a rewarding stimulus paired with sucrose solution (CS+) from a non-rewarding stimulus paired with quinine solution (CS−)^52^. Bees experienced 12 conditioning trials (6 CS+ and 6 CS− presentations in a pseudorandom sequence) in which only one stimulus was visible at a time, on either the right or left arm of the maze. In the opposite arm, a black screen was projected (Fig. 2a). At each trial, the visual target was presented during 30 s and paired with its corresponding reinforcement. Right after the choice of the CS+, the bees were allowed to feed from the sucrose solution *ad libitum* to promote their regular return to the setup from the hive. Thus, the inter-trial interval was set by the bee itself. Training was balanced so that for a group of bees the blue square and the green disc were the CS+ and the CS−, respectively, while for another group of bees the contingencies were reversed. Finally, we evaluated learning-induced preferences in a post-test presenting simultaneously the CS+ and the CS− in the absence of reinforcement (Fig. 2a).

#### Test performance: proportion of learners

**Figure 3:**
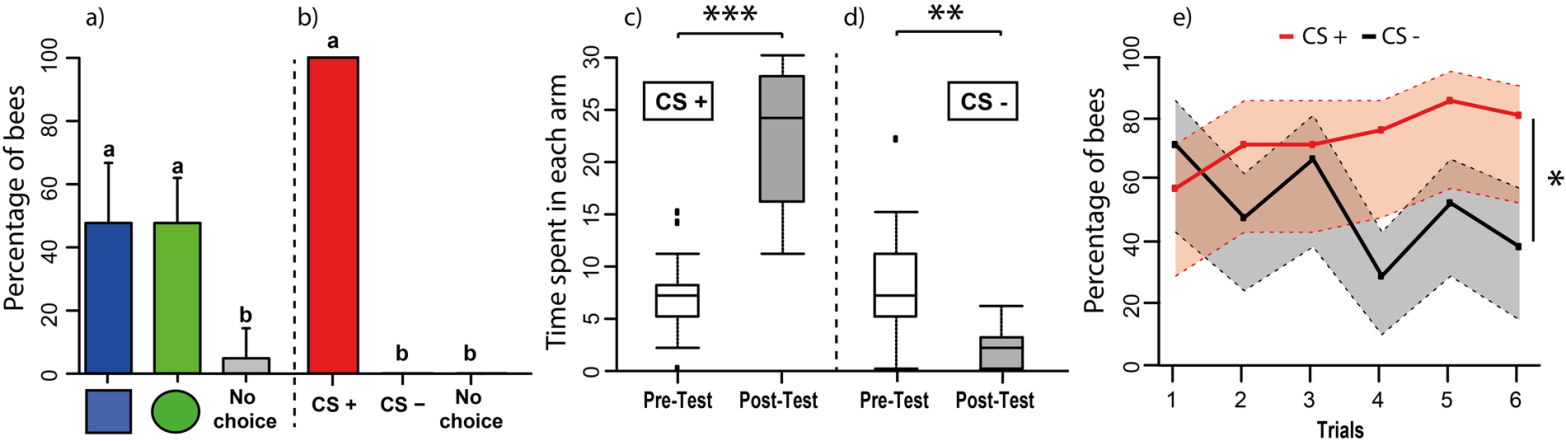
Visual learning of freely moving bees at the miniature maze [Experiment 1]. Left panels: Choice performance (percentage of bees choosing a given stimulus or not making a choice +95% confidence interval, based on the first choice made by the bees upon maze entrance) during the pre-test **(a)** and the post-test **(b)** at the mini Y-maze (n = 21). **(a)** Bars show the percentage of bees choosing spontaneously the blue square (blue bar), the green disc (green bar) or not choosing any stimulus (grey bar) during the pre-test. **(b)** Bars show the percentage of bees choosing the CS+ (red bar), the CS− (black bar) or not choosing (grey bar) after conditioning. Perfect learning was attained after conditioning as all bees chose the CS+ in the post-test. Different lower-case letters above bars indicate significant differences within each panel (p < 0.05). Middle panels: Time spent (median, quartiles and outliers) in each arm of the maze in the pre-test (white boxplot) and in the post-test (grey boxplot) during the 30 s of stimulus presentation. **(c)** Time spent in the CS+ arm. **(d)** Time spent in the CS− arm. Following conditioning, bees increased the time spent in the CS+ arm and concomitantly decreased the time spent in the CS− arm. **p < 0.001 ***p < 0.0001. Right Panel **(e)** **:** Acquisition performance (percentage of learners choosing the CS+, red curve, and the CS−, black curve; first choices) during the 12 conditioning trials (6 for each CS alternative) in the mini Y-maze. Bees (n = 21) decreased CS− responses while keeping CS+ responses high. Discrimination was significant. The 95% confidence interval is shown for each curve (dashed lines; in pink for the CS+ curve, and in grey for the CS− curve). *p < 0.05.

Spontaneous preferences between the blue square and the green disc were recorded during the pre-test, which lasted for the 30 s of stimulus presentation. The bees’ preference was recorded for all bees (n = 21) in terms of the first choice performed upon entrance into the mini maze. We calculated the percentages of bees choosing first the maze-arm corresponding to the blue square or the green disc, or not choosing any stimulus. In the pre-test, these percentages were 48%, 48% and 4%, respectively (Fig. 3a). There were no differences between the values corresponding to the choice of the blue square and of the green disc (GLMM binomial family with Tukey method for multiple comparison; blue square vs. green disc: z_124_ = 0, p = 1.00). The proportion of bees choosing either stimulus was significantly different from that of bees not choosing (blue square vs. no choice: z_124_ = 2.604, p = 0.025, green disc vs. no choice: z_124_ = 2.604, p = 0.025). After conditioning, all bees (100%; n = 21) exhibited perfect learning in the post-test (Fig. 3b). The totality of bees chose the CS+ and none of the bees chose the CS− or did not choose any stimulus in the maze. These results thus indicate that when bees had total freedom to choose video projected stimuli differentially reinforced, while walking in a dark environment, discrimination learning was fully successful.

#### Test performance: time spent in each maze arm during the pre-test and the post-test

In addition to the proportion of bees choosing either stimulus, we computed the time spent searching within arms displaying the CS+ or the CS− during the 30 s of stimulus presentation both during the pre-test and the post-test. During the pre-test, bees tended to spend a reduced proportion of the 30 s in the arms of the maze, probably due to their lack of familiarity with the setup (Fig. 3c,d; white box plots). At this stage, the time spent in the arms displaying the CS+ and the CS− did not differ (Wilcoxon test; U = 81, p = 0.51). After conditioning (Fig. 3c,d; grey box plots), bees increased the time spent in the CS+ arm and simultaneously decreased the time spent in the CS− arm (CS+ arm: U = 0, p < 0.0001; CS− arm: U = 183, p < 0.001). As a consequence, bees spent significantly more time in the CS+ arm than in the CS− arm (U = 0, p < 0.0001), thus showing a clear learning effect.

#### Acquisition performance

Bees experienced 6 CS+ and 6 CS− presentations in a pseudorandom sequence in which only one stimulus was visible at a time, on either the right or left arm of the maze. The bees’ performance was recorded in terms of the percentage of individuals choosing either the CS+ or the CS− when confronted against the black screen present in the alternative arm of the maze.

Bees showed a strong tendency to choose the single visual stimulus presented during the first trial, be it CS+ or CS− (Fig. 3e: ‘CS+, 57.2%; CS-, 71.4%’). Yet, a progressive differentiation in responses occurred during conditioning trials: while responses to the CS+ remained high (Fig. 3e, red curve), responses to the CS− decreased (Fig. 3e, black curve). This resulted in a non-significant CS effect (GLMM binomial family; df: 1, χ^2^ = 0.99, p = 0.32), a significant trial effect (df: 5, χ^2^ = 11.26, p = 0.046) and a significant interaction (CS*trial effect; df: 5, χ^2^ = 13.04, p = 0.023). These results reveal that bees learned the visual discrimination during training.

Finally, the proportion of time spent in each arm of the maze during the 30 s of CS display was also recorded during conditioning trials (Supplementary Fig. S1). During CS+ trials, bees spent almost the entire 30 s of CS+ display in the CS+ arm. In contrast, during CS− trials, bees tended to spend only one third of this time (ca. 10 s) in the CS− arm when the CS− was shown. These results show that during CS− trials, bees avoided choosing between two options, which did not yield the reward outcome they were searching.

Taken together, the results of this experiment show that freely flying bees trained to walk into a miniature Y-maze displaying video projected stimuli in a dark environment learn efficiently the visual discrimination when one of them is paired with sucrose and the other with quinine solution. This result sets, therefore, the basis for treadmill experiments in which a tethered, walking bee learns equivalent visual discriminations in a better-controlled visual environment.

### Experiment 2: visual learning at the treadmill

We determined if tethered honeybee foragers learn to discriminate the same two visual stimuli used in the previous experiment. Tethered bees (Fig. 1b) were placed on a treadmill in a dark room (Fig. 1c), which provided the same dark environment as in the mini-maze experiment. The setup was positioned in front of a semi-cylindrical screen of white tracing paper onto which visual stimuli were video projected (Fig. 1d). The projection background was the same as the one used in the Y-maze experiments. A set of mirrors was used to project the stimuli on the lateral parts of the semicircular screen (Fig. 1d) without deforming them. The visual stimuli appeared for 30 s on the screen and were 7 cm distant from the bee’s head. As in the mini-maze experiment, only one stimulus was shown at a time during conditioning, at 50° to either the left or right of the bee’s body axis (same angular position as in the maze experiment). The stimulus side was pseudorandomized from trial to trial (see Methods). Stimuli were equal in their total area (9 cm^2^). They subtended a similar visual angle to the bee eyes (square, from edge to edge: 24.2°; disc: 27.3°), which was consistent with that of the mini-maze experiment to ensure that chromatic vision was engaged for visual discrimination^50,51^. As in the maze experiment, stimulus intensity was adjusted to suppress a homogeneous spontaneous preference for the green disc uncovered by preliminary assays. The position of the visual stimuli was independent of the bees’ movements (open-loop condition) and did not vary during conditioning or testing. As stimuli were displayed at 50° on either side of the bee, a choice was counted if the cumulated rotation angle of the sphere exceeded 50° to the right or to the left during the 30 s of stimulus presentation. As cumulative rotation could exceed 360° during this measurement period, cumulative-heading values (°) were represented as multiples of 360°.

Three groups of bees were studied in parallel, interspersing bees from all three groups every day: a *paired group* (n = 38), a *CS (conditioned stimuli) group* (n = 32) and a *US (unconditioned stimuli) group* (n = 32). As in the mini-maze experiment, a pre-test assessed potential naïve preference for the green disc or the blue square presented simultaneously during 30 s in the absence of reinforcement (Fig. 2b). The percentage of bees choosing these stimuli or not choosing any stimulus (i.e. with a cumulative heading not exceeding 50° in either direction) and the cumulative heading of these bees were quantified during this period.

Subsequently, the *paired group* experienced a differential conditioning protocol (Fig. 2c) using positive and negative reinforcements as in the mini-maze experiment^52^. Bees in this group were trained with 12 conditioning trials spaced by one minute (Fig. 2b) in which one stimulus (CS+) was rewarded with 1 M sucrose solution while the other stimulus (CS−) was associated with a 60 mM quinine solution. The sucrose and quinine solutions were delivered to the proboscis by means of a toothpick if it was extended after stimulating the antenna on the side of the stimulus presentation. During training, only one stimulus (CS+ or CS−) was displayed on the screen in order to follow as closely as possible the procedure of the previous experiment with the maze (Fig. 2a,b). The sequence of CS+ and CS− was also pseudorandom (see Methods). Two subgroups were trained in order to balance reinforcement experience with the green disc and the blue square (i.e., one subgroup experienced the green disc rewarded and the blue square punished, while the other subgroup experienced the reversed contingency). The *CS group* experienced only visual stimulation (CS1, CS2) matched to that of the *paired group* (Fig. 2c), i.e. six green-disc and six blue-square presentations, but without any US. The *US group* experienced a sequence of 12 US presentations (6 sucrose and 6 quinine presentations) matching that of the *paired group* (Fig. 2c).

After the end of training, all three groups experienced a post-test in which bees were presented with the green disc and the blue square simultaneously, without any reinforcement (Fig. 2b). The percentage of bees choosing these stimuli or not choosing any stimulus and the corresponding cumulative headings were again quantified. In this way, performance in the post-test could be compared to that in the pre-test prior to training.

#### Test performance: proportion of learners

**Figure 4:**
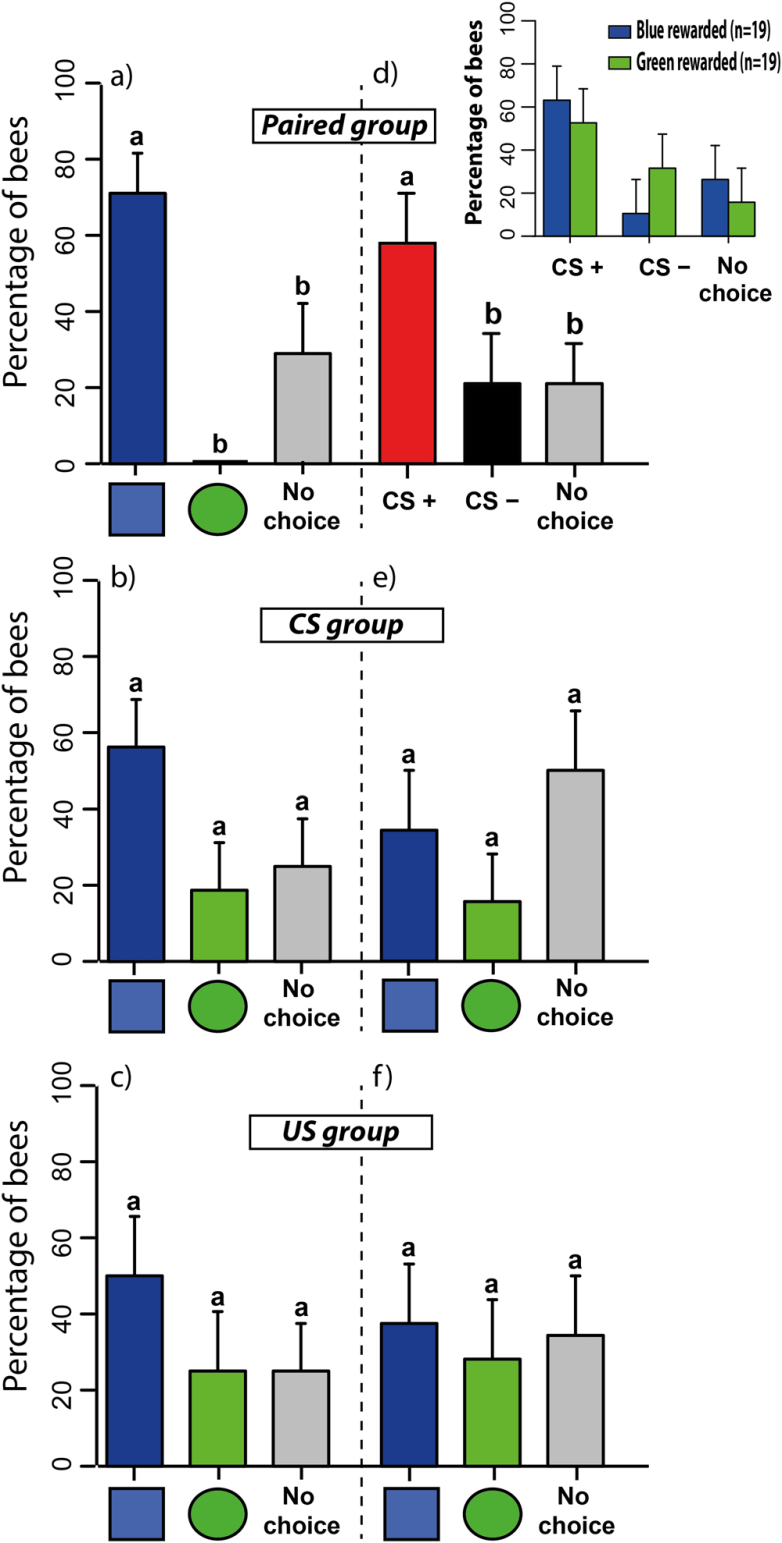
Spontaneous preferences (pre-test) and learning-induced preferences (post-test) at the spherical treadmill [Experiment 2]. Choice performance (percentage of bees choosing a given stimulus or not making a choice +95% confidence interval) during the pre-test (left panels) and the post-test (right panels). Upper graph: *paired group* (n = 38); middle graph: *CS group* (n = 32); *lower graph*: US group (n = 32). Bars in (**a**),(**b**) and (**c**) show the percentage of bees choosing spontaneously the blue square (blue bar), the green disc (green bar) or not choosing (grey bar) in the pre-test. The *paired group* showed a spontaneous preference for the blue square. Bars in (**d**) show the percentage of bees choosing the CS+ (red bar), the CS− (black bar) or not choosing (grey bar) after conditioning (paired group). Bars in (**e**) and (**f**) show the percentage of bees choosing the blue square, the green disc or not choosing in the *CS group* and the *US group*, respectively (during training, there were no reinforcements in the CS group and no visual stimuli in the US group). Only the *paired group* (**d**) exhibited a significant variation in the percentage of bees choosing between stimulus alternatives. In this case, the percentage of bees choosing the CS+ was significantly higher. The inset in (**d**) shows that this higher proportion was independent of the nature of the stimulus chosen as CS+ (blue square, n = 19, or green disc, n = 19). Different lower-case letters above bars indicate significant differences (p < 0.05).

We first focused on spontaneous choices in the pre-test. We quantified the percentage of bees that chose the green disc, the blue square or did not choose any stimulus (Fig. 4a,b,c). A significantly higher proportion of bees of the *paired group* (69%) preferred spontaneously the blue square (Fig. 4a). The proportion of bees choosing the green disc or not choosing any stimulus were 0% and 31%, respectively (GLMM binomial family with Tukey method for multiple comparison; blue square vs. green disc: z_915_ = 4.415, p = 0.0003, blue square vs. no choice: z_915_ = 4.52, p = 0.002, green disc vs. no choice: z_915_ = 2.10, p = 0.47). In the *CS group* (Fig. 4b), the proportion of bees spontaneously choosing the blue square was 56%, whereas 19% chose the green disc and 25% did not choose any stimulus. These proportions did not differ significantly from each other despite the tendency of bees to prefer the blue square (blue square vs. green disc: z_915_ = 2.98, p = 0.08, blue square vs. no choice: z_915_ = 2.49, p = 0.24, green disc vs. no choice: z_915_ = 0.60, p = 1.00). Finally, in the *US group* (Fig. 4c), the proportions of bees spontaneously choosing the blue square, the green disc or not choosing any stimulus were 50%, 25% and 25%, respectively. As for the *CS group*, these proportions did not differ significantly from each other despite the higher proportion of bees choosing the blue square (blue square vs. green disc: z_915_ = 2.03, p = 0.52, blue square vs. no choice: z_915_ = 2.03, p = 0.52, green disc vs. no choice: z_915_ = 0, p = 1.00). Consequently, a comparison between the three groups showed that they had different patterns of spontaneous responses (GLMM binomial family; group*choice effect: df: 4, χ^2^ = 10.58, p = 0.03). As the three groups were tested in parallel and experienced identical conditions in the pre-test, the significant spontaneous preference for the blue square in one of the three groups was likely due to a random sampling of individuals.

After the 12 trials experienced by each group after the pre-test, bees were again presented with the green disc and the blue square simultaneously in a post-test in the absence of reinforcement (Fig. 4d,e,f). The three groups differed in their stimulus choice during this post-test. In the *paired group* (Fig. 4d), a significant proportion of bees preferred the CS+ irrespective of the nature of the positively reinforced stimulus (blue square or green disc; see inset of Fig. 4d; reinforced-stimulus effect; df: 1, χ^2^ = 2.32, p = 0.12; reinforced-stimulus*choice effect: df: 2, χ^2^ = 3.37, p = 0.18). The absence of a significant reinforced-stimulus effect allowed pooling data from both subgroups (Fig. 4d). Globally, 60% of the bees preferred the CS+, while 20% preferred the CS− and another 20% exhibited no choice during the post-test (choice effect; df: 2, χ^2^ = 10.61, p = 0.005). These results thus show that learning occurred within the *paired group*.

For bees in the *CS group* (Fig. 4e), there was no reinforcement associated with the visual stimulation (i.e. there was neither a CS+ nor a CS−). Thus, the post-test performance was represented in terms of the proportion of bees choosing the green disc, the blue square or not choosing any stimulus (Fig. 4e). The same representation was adopted for the *US group* (Fig. 4f), which never experienced a CS stimulus but only positive and negative reinforcements on the left or right antenna corresponding to a theoretical presentation of a CS on the left or right of the screen, respectively, as performed in the paired group. The *CS group* did not show significant differences in the proportions of bees preferring either stimulus in the post-test (Fig. 4e). The proportion of bees spontaneously choosing the blue square was 34%, whereas 16% chose the green disc and 50% did not choose any stimulus. These values did not differ statistically from each other (blue square vs. green disc: z_1536_ = 1.69, p = 0.87, blue square vs. no choice: z_1536_ = −1.26, p = 0.98, green disc vs. no choice: z_1536_ = 2.80, p = 0.18). Similarly, in the *US group* (Fig. 4f), no significant differences were found between the bees preferring the blue square (38%), the green disc (28%) or not choosing (34%). These proportions did not differ from each other statistically (blue square vs. green disc: z_1536_ = 0.79, p = 1.00, blue square vs. no choice: z_1536_ = −0.26, p = 1.00, green disc vs. no choice: z_1536_ = 0.54, p = 1.00). Thus, neither the *CS* nor the *US group* varied their performance between the pre-test and the post-test (test effect; df: 1, χ^2^ = 3.01, p = 0.08; group effect; df: 1, χ^2^ = 0.07, p = 0.79; test*group effect; df: 1, χ^2^ = 0.28, p = 0.59). Only the training experienced by the *paired group* induced a significant increase in the proportion of bees preferring the CS+, which was particularly visible for bees rewarded on the green disc (compare Fig. 4a and inset in Fig. 4d).

#### Test performance: cumulative heading towards conditioned stimuli

**Figure 5:**
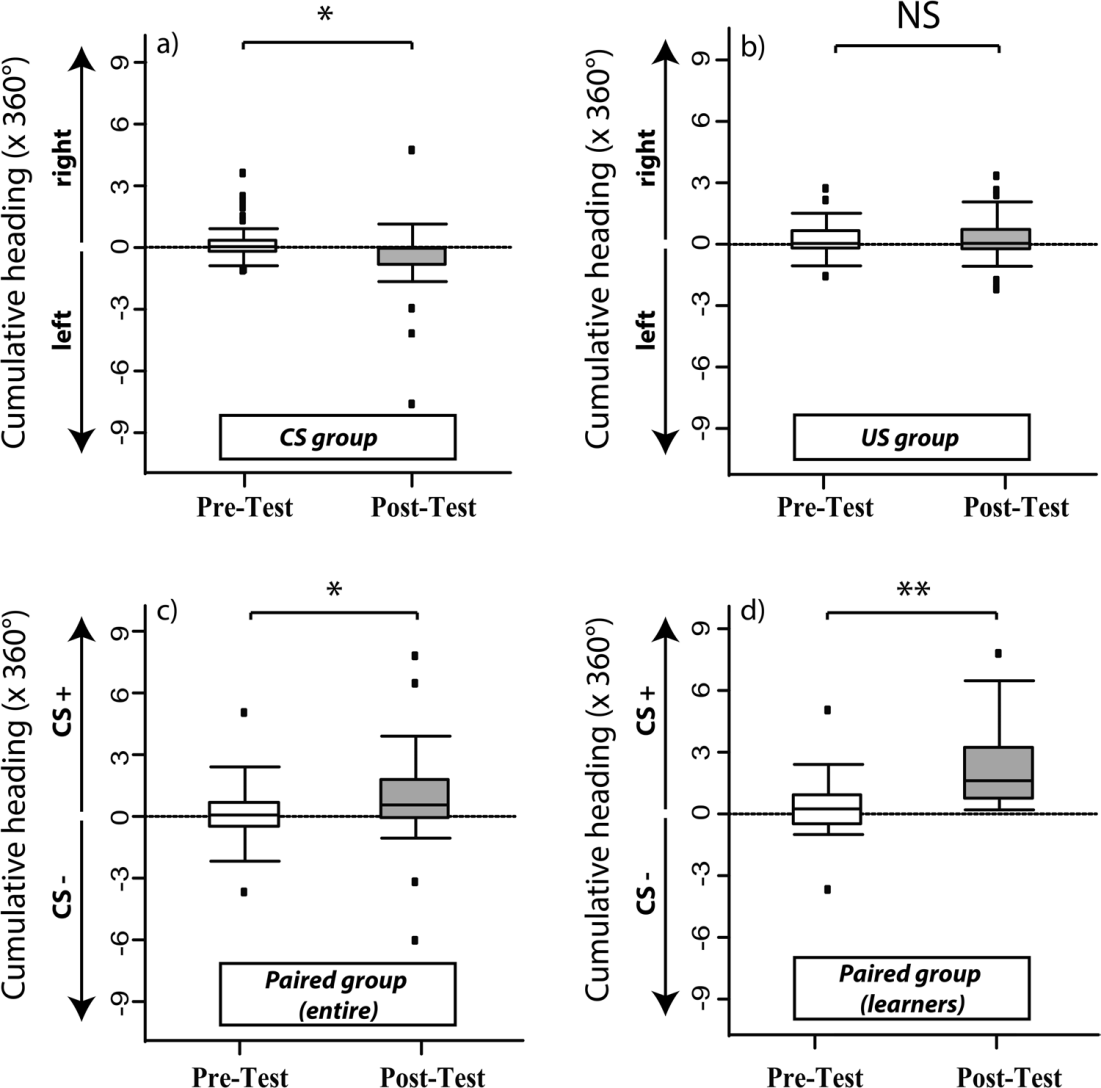
Cumulative-heading performance (in degrees; median, quartiles and outliers) during the pre-test (white boxplots, left) and the post-test (grey boxplots, right) at the spherical treadmill [Experiment 2]. Values represented correspond to multiples of a 360° rotation. (**a**) *CS group* (n = 32); (**b**) *US group* (n = 32); (**c**) *entire paired group* (n = 38); (**d**) Learners of the *paired group* (n = 22). In (**a**) and (**b**), as there was neither a CS+ nor a CS−, the convention is that the more positive was the cumulative heading, the more bees went to the right. In (**c**) and (**d**) the convention is that the more positive was this heading, the more bees chose the CS+. No variation in heading between pre-test and post-test was found in the *US group*; in the *CS group*, a tendency to turn to the left was visible after training. The paired group (entire and learners) oriented more towards the CS+ following conditioning. *p < 0.05; **p < 0.001; NS: non-significant.

These conclusions were confirmed by the quantitative analysis of the cumulative heading of the bees during the pre-test (Fig. 5, white box plots) and the post-test (grey box plots). Consistent with the absence of learning, bees of the *CS* (Fig. 5a) and the *US groups* (Fig. 5b) did not show any stimulus orientation during the post-test (*CS group*: U = 126, p = 0.08; *US group*: U = 325, p = 0.26). While bees of the *US group* did not change their heading direction between the pre-test and the post-test (Fig. 4b; U = 272, p = 0.89), bees of the *CS group* changed slightly their orientation and prioritized the left side (Fig. 4a; U = 375, p = 0.037), even if this did not induce any significant stimulus preference. In the case of the *paired group* (Fig. 5c), bees exhibited no stimulus preference during the pre-test (U = 461, p = 0.19), but oriented significantly towards the CS+ during the post-test after training (U = 567, p = 0.004). This change in orientation was significant (U = 223, p = 0.03) and confirmed that learning occurred within the *paired group*.

To determine to what extent this learning effect was masked by performance of non-learners in this group, we focused our analysis on bees that chose the CS+ during the post-test (Fig. 5d; “learners”; n = 22). During the pre-test, these bees did not show any stimulus preference (U = 169, p = 0.17) but headed significantly more towards the CS+ after training (U = 253, p < 0.001). This change in orientation was even more significant (U = 220, p = 0.002) than that of the entire *paired group*. Taken together, both the cumulative heading and the percentage of bees choosing the two stimulus alternatives during the post-test revealed significant visual learning in the *paired group* trained on the treadmill. This effect was neither apparent in the *CS* nor in the *US group*.

#### Acquisition performance

We next focused on the responses of the four groups of bees during their respective training schedules. For the *paired group*, the proportion of bees responding to the visual stimuli before US delivery was quantified. We evaluated both the entire group, as well as just the learners within the group. For the *CS group*, the same parameter was quantified, with the difference that these bees never experienced reinforcement associated with visual stimuli. Responses were thus evaluated during the CS period preceding a virtual US. For the *US group*, quantification of responses to CS stimuli was not possible as only reinforcement was delivered; nevertheless, we quantified responses occurring in the interval of time prior to US delivery on the left or right antenna, which corresponds to a theoretical CS stimulation on the left or right of the screen, respectively.

**Figure 6:**
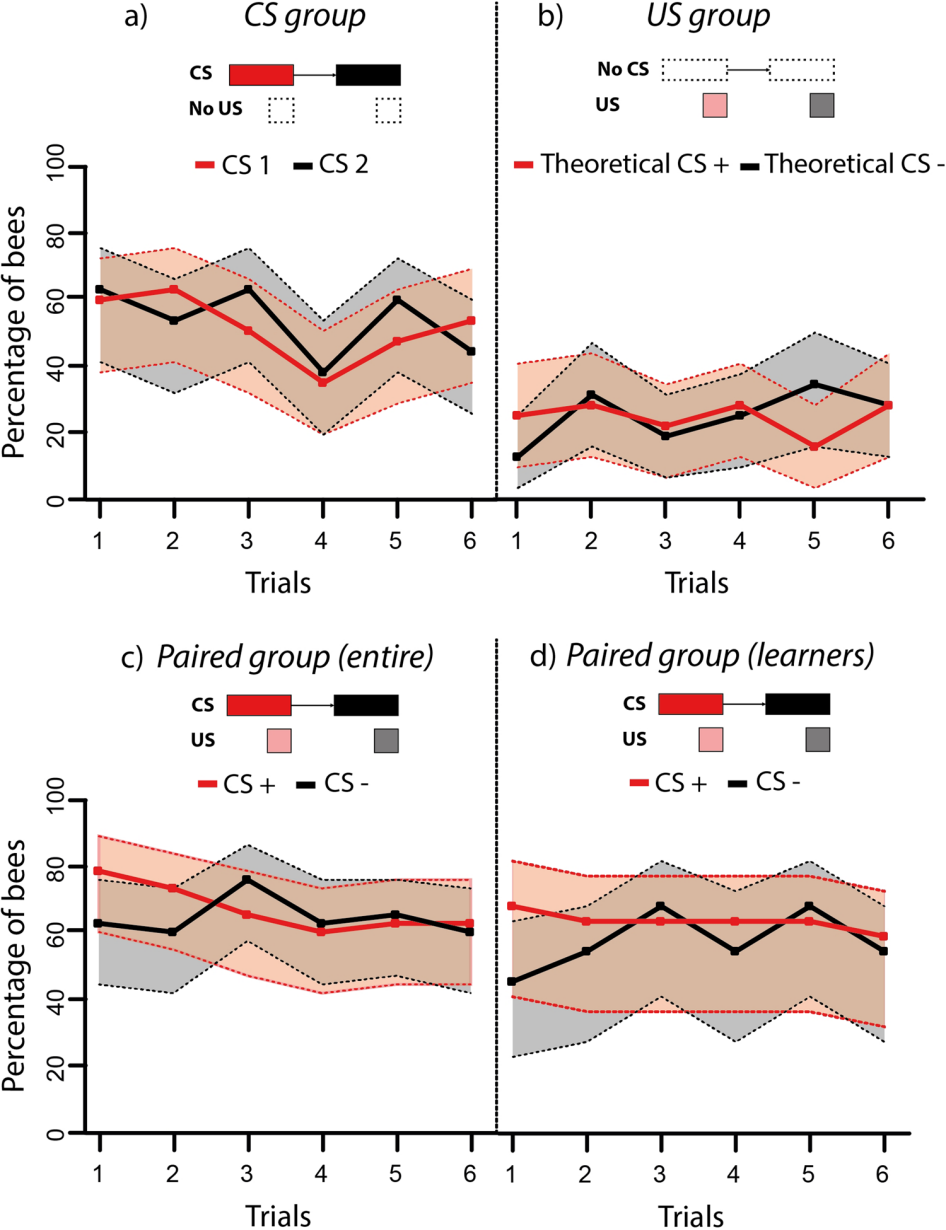
Acquisition performance (percentage of bees choosing the stimulus alternatives offered) during the 12 conditioning trials (6 for each alternative) at the spherical treadmill [Experiment 2]. (**a**) *CS group* (n = 32); this group received only visual stimulation without reinforcement (see inset) so that performance is represented in terms of bees choosing the CS1 (red curve) and the CS2 (black curve), which were presented in a succession matching that of the *paired group*. (**b**) *US group* (n = 32); this group did not receive visual stimulations (see inset) so that it is not possible to represent performance in terms of a CS response. The graph shows, therefore, the responses occurring in the interval of time prior to US delivery (red curve: responses to a ‘theoretical CS+’, prior to sucrose delivery; black curve: responses to a ‘theoretical CS−’, prior to quinine delivery). Reinforcement succession was matched to that of the *paired group*. (**c**) Entire *paired group* (n = 38) and (**d**) Learners of the *paired group* (n = 22); red curve: choice of the CS+ (paired with sucrose solution); black curve: choice of the CS− (paired with quinine solution). For all four groups, the 95% confidence interval is shown (dashed lines; in pink for the CS+, CS1 or theoretical CS+ curve, and in grey for the CS−, CS2 or theoretical CS− curve). No group showed significant discrimination between alternative stimuli.

Figure 6 shows the response curves obtained for the four groups during the 12 training trials. As expected from their test performances, neither the *CS group* nor the *US group* exhibited any discrimination (Fig. 6a,b). The *CS group* (Fig. 6a), which experienced a succession of green disc/blue square stimulations matching that of the *paired group* (CS1/CS2), did not respond preferentially to any stimulus during the 12 trials (CS effect; df: 1, χ^2^ = 0.15, p = 0.69). Yet responses decreased similarly along trials for both stimuli (trial effect; df: 5, χ^2^ = 14.63, p = 0.01; CS*trial effect; df: 5, χ^2^ = 6.90, p = 0.23), which indicates that in the absence of reinforcement, bees responded progressively less to the green disc and the blue square, a phenomenon akin to habituation. The *US group* (Fig. 6b) did not respond preferentially during the interval preceding the sucrose solution (‘theoretical CS+’) or the quinine solution (‘theoretical CS−), simply because they had no predictive cue enabling this discrimination. In this case, their response along trials remained invariable (CS effect; df: 1, χ^2^ = 1.90, p = 0.16; trial effect; df: 5, χ^2^ = 6.68, p = 0.24; CS*trial effect; df: 5, χ^2^ = 5.77, p = 0.33). Interestingly, the performance of the entire *paired group* (Fig. 6c) was similar to those of the *CS* and *US groups* as no discrimination was visible along trials (CS effect; df: 1, χ^2^ = 3.08, p = 0.08; trial effect; df: 5, χ^2^ = 4.06, p = 0.54; CS*trial effect; df; 5, χ^2^ = 6.26, p = 0.28). Such an absence of discrimination could have been due to a potential masking of the learners’ performance by that of non-learners in the entire *paired group*. To examine this possibility, we restricted the analysis to learners. The resulting acquisition curves (Fig. 6d) were surprisingly similar to those of the *CS group* and of the entire *paired group*, thus revealing an apparent absence of discrimination (CS effect; df: 1, χ^2^ = 3.37, p = 0.07; trial effect; df: 5, χ^2^ = 5.19, p = 0.39; CS*trial effect; df; 5, χ^2^ = 3.46, p = 0.63). Yet, as shown by post-test performances, bees of the *paired group* (entire group and learners) had learned the visual discrimination.

A strong tendency to choose the single visual stimulus presented during the first trial, be it CS1 or CS2, or CS+ or CS−, was clearly visible both in the *CS* and in the *paired groups* (Fig. 6a: *CS group*; CS1: 56%, CS2: 59%; Fig. 6c: entire *paired group*; CS+ : 76%, CS−: 61%; Fig. 6d: learners of the *paired group*; CS+ : 68%, CS−: 45%). This tendency was similar in the first trial of all three groups (all between-group comparisons NS) and occurred before any reinforcement delivery in the *paired* groups. This shows that bees were responding to the mere presence of the first visual stimulus displayed on the screen and simply kept responding alternately to the CS+ and the CS− during successive trials. This non-specific CS attraction suggests that positive phototaxis and/or object fixation drove the bees towards the visual stimulus displayed on the screen during a training trial and were the cause for the similar performance of the *CS* and the *paired groups*. The *US group* (Fig. 6b) showed a level of responses which was significantly lower than that of the other three groups during the 12 trials (group effect; χ^2^ = 9.30, df: 3, p = 0.02; *paired (entire) vs*. *US group*: z_5948_ = 11.51, p < 0.0001; *paired (learners)* vs. US group: z_5948_ = 8.5, p < 0.0001; *CS vs*. *US group*: z_5948 = _7.27; p < 0.0001). This confirms that responses during training of the *CS* and the *paired groups* were driven by object fixation and/or positive phototaxis, two components that were absent in the *US group*, which did not experience any visual stimulus.

An analysis of the mean cumulative heading angle during the conditioning phase performed for all groups (Supplementary Fig. S2a,b,c,d) confirmed the presence of a strong positive phototaxis or object fixation in bees of the *CS group* (Supplementary Fig. S2a) and of the *paired groups* (entire group: Supplementary Fig. S2c; learners: Supplementary Fig. S2d). These bees headed towards the visual stimulus presented (Supplementary Fig. S3), independently of its contingency (in the case of the *paired group*; i.e. they headed towards the CS+ or the CS− when these stimuli were presented alternately during training), its nature (in the case of the *CS group*, i.e. they headed towards the green disc or the blue square) or its position (right or left). By contrast, the bees from the *US group* (Supplementary Fig. S2b) which were not attracted by visual stimulation, walked in a rather straightforward way but in the absence of visual stimulation, they tended to walk less.

We thus conclude that presentation of a single-color stimulus during training induces strong phototaxis or object fixation at the treadmill. This confounding factor impedes measuring acquisition of a visual discrimination in our controlled conditions, even if a significant percentage of bees in the *paired group* learned the associations between visual stimuli and reinforcement. Thus, in addition to the clear evidence for learning found in the *paired group* during the tests with simultaneous presentation of trained stimuli, phototaxis and object fixation are also factors that need to be considered under certain training regimes.

### Experiment 3: the influence of negative unconditioned stimuli on visual learning at the treadmill

An important topic in associative learning studies relates learning strength to the relative strength of the US in terms of its ability to promote conditioning to the CS^53^. In this experiment, we aimed at determining which aversive US had a higher relative strength to promote visual conditioning in a joint manner with the appetitive sucrose US. In differential olfactory PER conditioning, concentrated saline solution is more efficient than quinine solution as a negative US^54^. Here we asked if changing the nature of the US associated with the CS− also modulates learning success at the treadmill.

We studied the performance of four groups of bees that experienced the conditioning schedule of the *paired group* of the previous experiment. All groups experienced 1 M sucrose solution as the US paired with the CS+, but differed in the nature of the US associated with the CS−, which could be 60 mM quinine (n = 32), 3 M NaCl (n = 32), distilled water (n = 33) or contact with a dry toothpick (n = 32). The *quinine group* therefore reproduces the treatment experienced by the *paired group* of the previous experiment (Experiment 2).

#### Test performance: proportion of learners

**Figure 7:**
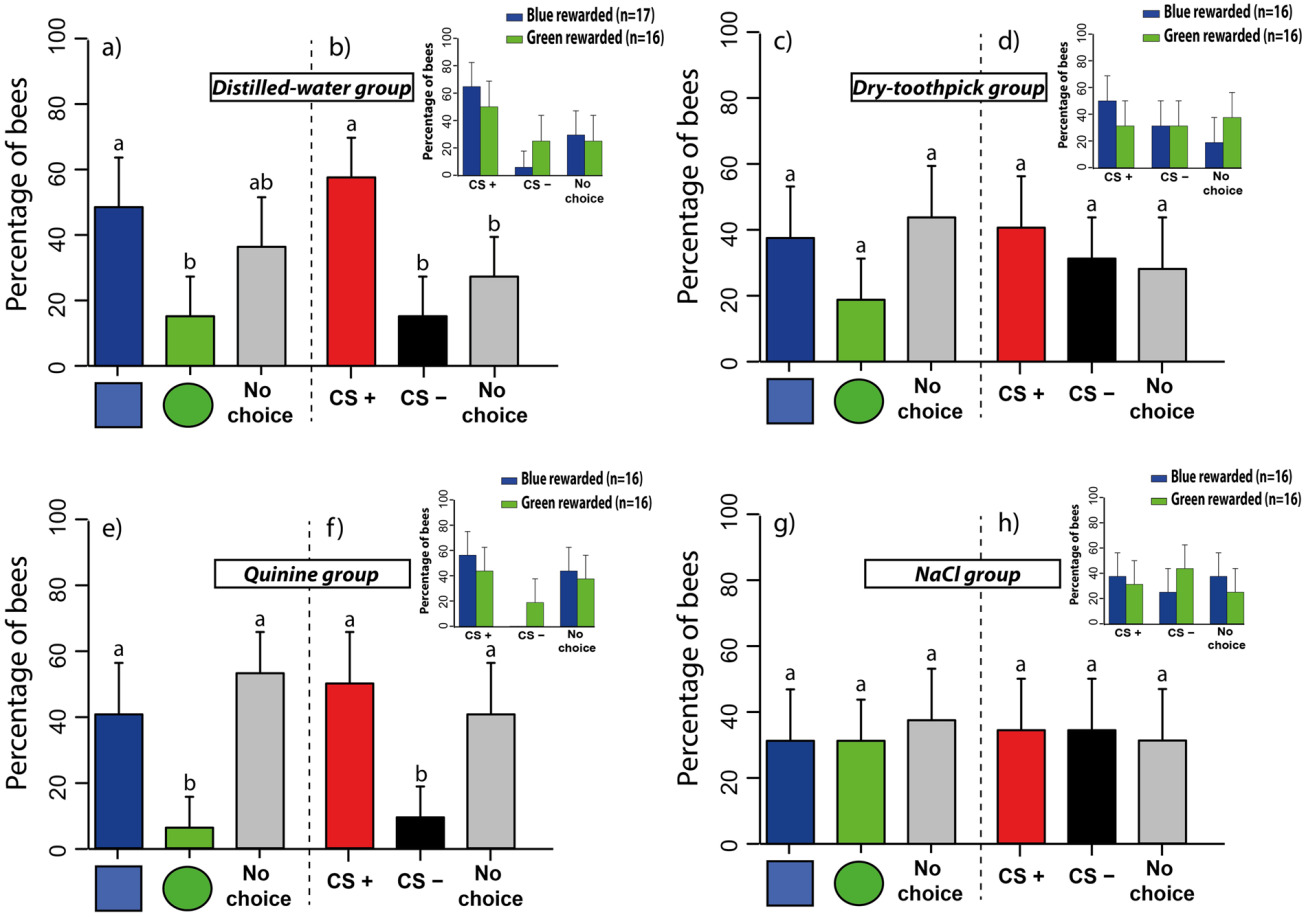
Spontaneous preferences and learning-induced preferences at the spherical treadmill for groups, which got different negative unconditioned stimuli (US) on the CS− [Experiment 3]. The graph shows the choice performance (percentage of bees choosing a given stimulus or not making a choice +95% confidence interval) during the pre-test (left panel in each graph) and the post-test (right panel in each graph). (**a**,**b**) *distilled-water group* (n = 33); (**c**,**d**) *dry-toothpick group* (n = 32); (**e**,**f**) *quinine group* (n = 32); (**g**,**h**) *NaCl group* (n = 32). Bars in (**a**,**c**,**e**,**g**) show the percentage of bees choosing spontaneously the blue square (blue bar), the green disc (green bar) or not choosing (grey bar) in the pre-test. The *quinine group* and the *distilled-water group* had a higher proportion of bees preferring the blue square compared to bees preferring the green disc. Bars in (**b**,**d**,**f**,**g**) show the percentage of bees choosing the CS+ (red bar), the CS− (black bar) or not choosing (grey bar) after conditioning. In both the *quinine group* and the *distilled-water group* a higher proportion of bees preferred the CS+ compared to bees preferring the CS−. The insets in (**b**) and (**f**) show that this higher proportion was independent of the nature of the stimulus chosen as CS+ (blue square or green disc). Different lower-case letters above bars indicate significant differences (p < 0.05).

In the pre-test (Fig. 7a,c,e,g), the *distilled water group* showed a spontaneous preference for the blue square (Fig. 7a). The proportions of bees spontaneously choosing the blue square, the green disc or not choosing any stimulus in this group were 49%, 15% and 36%, respectively (blue square vs. green disc: z_392_ = 2.78, p = 0.014, blue square vs. no choice: z_392_ = 1.00, p = 0.58, green disc vs. no choice: z_392_ = 1.92, p = 0.13). In the *dry-toothpick group* (Fig. 7c), these proportions were 38%, 19% and 44%, respectively (blue square vs. green disc: z_380_ = 1.64, p = 0.23, blue square vs. no choice: z_380_ = −0.51, p = 0.87, green disc vs. no choice: z_380_ = 2.11, p = 0.08). In the *quinine group* (Fig. 7e), the proportions of bees choosing the blue square, the green disc or not choosing any stimulus were 41%, 6% and 53%, respectively (blue square vs. green disc: z_380_ = 2.86, p = 0.01, blue square vs. no choice: z_380_ = −0.99, p = 0.58, green disc vs. no choice: z_380_ = 3.49, p = 0.001). Finally, in the *NaCl group* (Fig. 7g), these proportions were 31%, 31% and 38% respectively (blue square vs. green disc: z_380_ = 0, p = 1.00, blue square vs. no choice: z_380_ = −0.53, p = 0.86, green disc vs. no choice: z_380_ = 0.53, p = 0.86). A comparison between all four groups did not reveal significant differences (group effect: χ^2^ = 0.70, df: 3, p = 0.87; choice effect, including both CSs and no-choice: χ^2^ = 4.73, df: 2, p = 0.09; CS effect, including both CSs only: χ^2^ = 0.57, df: 1, p = 0.44; group*choice effect: χ^2^ = 3.96, df: 6, p = 0.68; group*choice*CS effect: χ^2^ = 7.84, df: 11, p = 0.72). The fact that the choice effect was close to significance was probably due to the *distilled water group* and the *quinine group*, in which more bees preferred significantly the blue square in the pre-test.

After conditioning (Fig. 7b,d,f,h), the performance in the post-test was independent of the stimulus (blue square or green disc) chosen by the experimenter as CS+ or CS− (CS effect: χ^2^ = 0.57, df: 1, p = 0.44; group*choice*CS effect: χ^2^ = 7.68, df: 11, p = 0.74, see insets in Fig. 6e,f,g,h). Thus, for each group, performance was represented in terms of the proportion of bees choosing the CS+, the CS− or not choosing any option. In the *distilled water group* (Fig. 7b), a significant learning effect was found: 58% of the bees preferred the CS+, while 15% preferred the CS− and 27% exhibited no choice during the post-test (CS+ vs. CS−: z_392_ = −3.38, p = 0.002, CS+ vs. no choice: z_392_ = 2.44, p = 0.03, CS− vs. no choice: z_392_ = −1.19, p = 0.46). In the dry-*toothpick group* (Fig. 7d), no variation was found in the proportion of bees choosing the CS+, the CS− or not choosing any option. Percentages for these three categories were 41%, 31% and 28%, respectively, and did not differ from each other (CS+ vs. CS−: z_380_ = −0.78, p = 0.71, CS+ vs. no choice: z_380_ = 1.05, p = 0.55, CS− vs. no choice: z_380_ = 0.27, p = 0.96). In the *quinine group* (Fig. 7f), a significant learning effect was found as 50% of the bees preferred the CS+, while 9% preferred the CS− and another 41% exhibited no choice during the post-test (CS+ vs. CS−: z_380_ = −3.23, p = 0.004, CS+ vs. no choice: z_380_ = 0.75, p = 0.73, CS− vs. no choice: z_380_ = −2.68, p = 0.02). Finally, in the *NaCl group* (Fig. 7h), no significant learning was found as the proportions of bees choosing the CS+, the CS− or not choosing any option were 34%, 34% and 32%, respectively (CS+ vs. CS−: z_380_ = 0, p = 1.00, CS+ vs. no choice: z_380_ = 0.27, p = 0.96, CS− vs. no choice: z_380_ = 0.27, p = 0.96). A comparison between all four groups showed no differences (group effect: χ^2^ = 2.94, df: 3, p = 0.40; choice effect: χ^2^ = 4.73, df: 2, p = 0.09; group*choice effect: χ^2^ = 3.96, df: 6, p = 0.68). This was confirmed by a Tukey test for multiple comparisons between proportions, which allowed comparing the proportion of learners between all four groups (*distilled-water group*: 19/33 learners; *quinine group:* 16/32 learners; *dry-toothpick group:* 11/32 learners; *NaCl group*: 13/32 learners; q_∞,4_ < 3, 633 for all comparisons, NS). However, significant differences in favor of the CS+ were only found in the post-test of the *quinine* and the *distilled-water groups* while no differences between choice categories were found in the post-test of the *toothpick* and the *NaCl groups*. This difference indicates that learning occurred only in the *quinine* and *distilled groups*, in which more CS+ choosers were found as a consequence of conditioning, but not in the *toothpick* and *NaCl groups* where bees distributed equally between CS+ choosers and CS− choosers in the post-test. Thus, quinine solution and distilled water were more efficient as negative US and supported discrimination learning while contact with the dry toothpick or the 3 M NaCl solution did not.

#### Acquisition performance

The analysis of the acquisition curves revealed again a phototactic/object fixation effect similar to that found in Experiment 2. Bees in all groups exhibited a strong tendency to choose the single visual stimulus presented, be it CS+ or CS−, from the very first trial and kept doing so during the 12 trials. Focusing on learners in all four groups did not change this conclusion (Supplementary Fig. S4a,b,c,d), thus showing that either phototaxis or object fixation overshadowed learning, as in the previous experiment. An analysis of the mean cumulative heading angle of learners during conditioning confirmed this conclusion: despite exhibiting a significant cumulative heading towards the CS+ during the tests, learners in all four groups did not show any evidence of learning during the 12 conditioning trials when the same behavioral parameter (cumulative heading) was considered (Supplementary Fig. S5a,b,c,d).

We thus concluded that presenting a single training stimulus at a time overshadows learning in the treadmill. Learning occurred in all four paired groups but not all US associated with the CS− yielded the same learning success. Indeed, quinine and distilled water were more effective as negative US as in these cases significantly more learners were found.

## Discussion

We aimed at establishing the bases for the study of visual learning in honeybees in a controlled laboratory environment allowing future explorations of its neural underpinnings and the use of virtual reality to this end. We tethered individual bees and allowed them to walk on a treadmill while displaying visual stimuli in front of them by means of a video projector. The ultimate goal was to reproduce visual learning performances by free-flying bees^8,16^, while partially constraining the freedom of movement. Such a constraint is necessary to allow the simultaneous study of the neural architectures underlying different forms of visual learning, a goal that has proven elusive in bees until now. Our results show that bees walking into a miniature maze set in a dark environment successfully learn to discriminate visual stimuli projected onto the back walls of the maze (Experiment 1). Adapting this experimental situation to a treadmill setup (Experiment 2) showed that learning performances were better in the maze, i.e. that full movement freedom and behavioral context are important for visual learning. Yet, robust visual learning occurred at the treadmill, thus showing that it is possible to reproduce elemental forms of visual learning in this setup. Despite the experimental constraints imposed by tethering and by setting the bees in a dark surrounding, a significant percentage of animals exhibited discrimination learning performances and preferred, after training, the visual stimulus associated with sucrose reward (CS+) over a negative stimulus (CS−). We also show that the nature of the US associated with the CS− affects the learning performance (Experiment 3). Indeed, quinine and distilled water were more effective as in these cases significantly more bees learned the visual discrimination. These results set the bases for further explorations and refinements of the protocols described here.

The learning success (proportion of learners) at the treadmill varied between 50% and 60% (Experiments 2 & 3). These values are non-negligible compared to the lower percentage of learners (usually, between 30% and 40%) that are reported in experiments on visual PER conditioning^55–58^. Full immobilization is imposed by PER conditioning, contrary to our experimental situation in which bees walk stationary on the treadmill. Yet, although the results obtained in our work are promising for further explorations of visual learning, they seem to be distant from the 100% found in the mini maze (Experiment 1) and usually reported in visual learning experiments with free-flying bees^8,10,16^. Note, however, that studies on visual learning in freely flying bees usually discard individuals that for some reasons do not learn the task (e.g. bees that do not return to the experimental site and that do not complete, therefore, the training, or bees that develop a tendency to turn always to the same side in a maze). Had these works included a thorough analysis of non-learners, as in this work, the proportion of success under the two situations - treadmill and free flight - could be perhaps more similar.

It seems, nevertheless, that the critical factor for successful visual learning is that bees dispose of a certain freedom of movement, as shown by Experiment 1. It could be argued that the lack of freedom affects the animal’s appetitive motivation, thus decreasing performance success in protocols that do not grant such freedom. Yet, this argument can be discarded by considering that olfactory PER conditioning, in which bees are fully immobilized, yields a learning success that typically reaches 90–100%^2,11,12^. Thus, if a decrease in motivation is not a likely explanation for the decrease in learning success observed when movement is restricted, what could be the cause for such a learning impairment? We suggest that the key factor is the impaired possibility for exerting active scanning while learning a visual task. In active vision, animals vary the viewpoint in order to investigate the environment and extract better information from it. Honeybees scan visual scenes sequentially, rather than being able to taking them in “at a glance”^59,60^. This need for serial scanning means that shape differences can only be identified with difficulty if stimuli are presented for short intervals, or when bees cannot move their eyes, as happens when they are fully restrained. Performing flight maneuvers allows extracting and acquiring information about the nature and structure of visual targets^44,61,62^. Bees, flies and other flying insects actively shape the dynamics of the image flow on their eyes, a strategy that facilitates the solving of spatial vision tasks in an efficient way. In this context, active vision is crucial to segregate rotational from translational optic flow components. Stereotyped circling or scanning flights are used to this end^24,44–47^, which help, in the case of 2D stimuli like the ones used in our work, to extract the borders of objects for better recognition^48,49,63^. An alternative explanation may put the accent on the difference in behavioral contexts between the treadmill and the mini maze. In the mini-maze, the bees flew back to the hive between trials and returned to the setup on their own accord, conditions which might facilitate associative learning performances. In the treadmill, on the contrary, bees were not free to choose when to enter the experimental trials, and were not able to return to the hive between trials. Note that these limitations also apply to PER experiments, which do not affect efficient olfactory learning and memory formation. Yet, it could be that olfactory learning is less sensitive to these aspects than visual learning.

Both the stimuli displayed by the video projector and the illumination conditions were the same in the miniature maze and in the treadmill setup. The mini-maze used in Experiment 1 certainly imposed a differently structured environment (e.g., maze arms vs open visual field at the treadmill) but the visual stimuli were in the same angular range as in the treadmill. In addition, the associative framework occurring in both scenarios had comparable components: learning in the Y-maze experiment was mainly operant, since reinforcement outcome depended on the bees’ choice^64^. Yet, as in most operant protocols using discriminative stimuli, learning also included Pavlovian components as the bees learned the association between a given visual stimulus (conditioned stimulus, CS) and reinforcement/punishment (unconditioned stimulus, US). In the treadmill experiments, there were also obvious Pavlovian components as the CS+ and the CS− (and their respective US at the end of each CS) were delivered in a pseudorandom yet fixed sequence for every bee. Yet operant associations cannot be excluded due to the strong tendency of bees to walk towards the single illuminated stimulus displayed during training, which resulted in the delivery of an appetitive or an aversive US. In other words, associations between walking towards a CS+ and the appetitive US, or between walking towards a CS− and an aversive US, could also occur, despite the fixed sequence of presentation of the CS+ and CS− and their respective US, over which bees had no control.

A main difference between the maze and the treadmill scenarios lies in the possibility of moving and actively scanning the visual targets, and of seeing subsequent changes in stimulus properties following this active sensing. These features may account for differences between these experimental contexts. In the maze, scanning and visual stimulus control were possible while they were reduced and/or inexistent in the treadmill, in particular because of the open loop conditions imposed on the bees. Consequently, no change in stimulus perceptual properties occurred following the bee’s translation on the treadmill. Thus, performing these or other experiments under closed-loop conditions in which the projected stimuli update in real time in response to the bee’s movements could be crucial to improve learning success.

Our original discrimination protocol used 1 M sucrose solution as positive US paired with the CS+, and 60 mM quinine solution as a negative reinforcement paired with the CS−. In doing this, we aimed at reproducing conditions that have been used repeatedly in visual learning experiments with free-flying bees^52,65^ and that were suggested to enhance attentional mechanisms and improve discrimination performances^66^. Consistent with our findings, prior studies showed that not all aversive US have the same consequences for discrimination learning. Free-flying bumble bees trained to discriminate between two perceptually similar colors, one associated with 1.75 M sucrose solution, and the other with water or quinine solution 120 mM, perform better if they experience quinine on the CS− targets rather than water^65^.

In differential conditioning, the higher the contrast between the subjective value of the CS+ and the CS−, the better the learning. In the case of the toothpick (Experiment 3), the antennal stimulation was purely mechanical and did not generate an appetitive expectation. This stimulus did not offer, therefore, a marked contrast with the appetitive sucrose solution experienced upon CS+ presentation. It is thus understandable that the toothpick stimulation did not induce a higher proportion of learners. In the case of quinine solution and distilled water, the animals experienced an aqueous solution on the antennae which induces in the first trial PER, followed by contact of the proboscis with these solutions. Here the contrast between the CS+ and the CS− existed in terms of expectation and real outcome as bees extended the proboscis expecting an appetitive stimulus and experienced water or quinine, which do not correspond to their expectancy. The fact that the results were similar for quinine solution and water may be explained by prior works indicating that bees have a reduced sensitivity to bitter substances at the level of their antennae and may thus detect an aqueous solution rather than a bitter solution^67^. The fact that until now, no bitter-taste receptor genes have been identified in the honey bee genome^68^, contrary to other insects, strengthens this conclusion. It has, therefore, been suggested that the aversive effect of concentrated quinine solution could have two main explanations: (1) in the long delay, a malaise effect induced by the consumption of this substance^69,70^; (2) in the short delay, the successive negative contrast experienced by a bee searching for sucrose solution and receiving instead a watery solution that does not fulfill its appetitive expectation^71^. The fact that we found the same improvement with distilled water and 60 mM quinine solution supports the second explanation. It is, nevertheless, intriguing that a concentrated NaCl solution paired with the CS− did not support learning. Indeed, in olfactory discriminations with harnessed bees, this solution induced better learning performances (i.e. had more aversive strength) than the same quinine solution used in our experiments^54^. Further studies should explore why NaCl did not have the expected aversive strength in our experimental conditions.

In Experiments 2 and 3 performed at the treadmill, the acquisition curves for tethered bees did not reveal successful discrimination learning (see Fig. 6 and Supplementary Fig. S4) even if such learning occurred and was afterwards visible in the post-test performances (see Figs 4, 5 and 7). This was due to a confounding effect of either phototaxis or object fixation, which led bees to choose the single illuminated visual target that was displayed at a time on the projection screen, irrespectively of its nature and reinforcement. During successive trials, bees kept choosing the single stimulus that was shown in a given trial, thus masking discrimination learning. Setting the setup in darkness was necessary to display salient stimuli using the video projector. This could have enhanced phototactic tendencies but Experiment 1 offered the same illumination conditions in the mini maze, and although a phototactic/object fixation effect was seen in the first trial (see Fig. 3e), it did not overshadow the bees’ discrimination learning. Thus, darkness was not the primary cause for phototactic/object-fixation overshadowing of learning during training at the treadmill. The reason for this effect is to be found in our training schedule. By offering a single illuminated stimulus in any conditioning trial, bees were compelled to orient towards it. In Experiment 1, when an equivalent situation was offered in CS− trials in the mini maze, bees decided not to enter the maze arm in almost half of the time of CS− display (Supplementary Fig. S1), yet in this case they had the freedom to do so. Thus, reconfiguring the training in order to display both the CS+ and the CS− in any conditioning trial should decrease both spontaneous phototaxis and object fixation, and force the animals to choose between alternatives with different reinforcement outcomes. The simultaneous presentation of both the CS+ and the CS− during training requires closed-loop conditions, in which responses are tracked and used to update the next ‘view’ of the virtual environment in real time. These conditions allow the bee to ‘move’ the stimulus chosen towards her and thus to improve the nature of the associations established upon reward or punishment delivery during training.

We conclude that visual learning in honey bees is amenable to a laboratory preparation in which tethered animals learn visual stimuli in a controlled visual environment. This result is important for further analyses of the neural bases of such learning, which have remained poorly explored until now. We have identified potential caveats and suggested further improvements for future studies under these experimental conditions: simultaneous presentation of the CS+ and the CS− during training, stimulus display under closed-loop conditions, careful choice of the reinforcement associated with the CS− and enhancement of active vision, for instance through 3D stimulus display, are possible ways to improve learning performances in our setup. Such experiments will allow us to learn more about the mechanisms of visual learning and perception in the honey bee, thus reinforcing the model status of this insect for this research field.

## Methods

Honey bees (*Apis mellifera*) were obtained from the apiary located at the campus of the University Paul Sabatier. Only foragers caught upon landing on a gravity feeder filled with a 0.9 M sucrose solution were used in our experiments to ensure high appetitive motivation.

### Experiment in the mini-maze

Bees were fully free to fly from the hive to the laboratory where they approached and entered the mini-maze to collect sucrose solution. Bees were previously trained to fly to a gravity feeder filled with 0.9 M sugar water where they were marked with acrylic paints on the thorax and/or abdomen for individual identification. A single marked bee was randomly chosen from the feeder and moved progressively to the mini-maze by means of an Eppendorf tube cover offering sucrose solution. Similar small mazes have been used to successfully train walking bees to collect sucrose solution^54,72^. The maze was placed in a dark room, with illumination conditions that were similar to those set for the treadmill. Each bee was trained systematically to enter the Y-maze by itself in order to find sucrose solution at the small feeder. No visual stimulus was offered during this pre-training phase. The reduced size of the maze prevented the bee from flying. It could nevertheless freely walk inside the maze arms to search and collect the sucrose solution. Only one bee at a time was present inside the maze.

#### The mini Y-maze

The maze (Fig. 1a) consisted of three arms shaped in a Y. Each arm had a length of 5 cm and a cross-section of 3 × 3 cm. The visual stimuli were projected by a video projector (Acer K135i, Roissy, France; Fig. 1c) onto tracing-paper screens placed vertically at the ends of the two arms. The stimuli were, therefore, at 5 cm from the decision point of the maze (arm intersection). Small Eppendorf tube covers (Eppendorf tube 3810x, Hamburg, Germany) containing 60 µl of 1 M sucrose solution or 60 mM quinine solution were placed at the end of the arm presenting the respective visual stimulus. The setup was placed under a red ceiling to ensure a dark environment for the bees.

#### Visual stimuli

The visual stimuli to be discriminated were a blue square (RGB: −1, −1, 1) and a green disc (RGB: −1, 0.2, −1). The blue square was 2.34 × 2.34 cm and the green disc was 2.64 cm in diameter. Their intensity was 14000 µW/cm^2^ for the blue square and 1800 µW/cm^2^ for the green disc. Both stimuli displayed the same total area (5.5 cm^2^). They subtended a similar visual angle to the bee eyes (square, from edge to edge: 26.3°; disc: 29.6°), which was in the same range as those presented at the treadmill. This angular range ensures that bees engaged their chromatic vision to achieve the visual discrimination^50,51^.

#### Conditioning and testing procedure

The conditioning and testing procedures (Fig. 2a) mirrored as closely as possible those used at the treadmill (Experiment 2, paired group; Experiment 3, quinine group; see below). The bees were first trained to forage on an external feeder, which was progressively moved close to the mini maze inside the dark room. A single marked bee was trained gradually to enter the maze to collect sucrose solution. No visual stimulus was presented therein. Once the bee entered the maze by itself four consecutive times (i.e. after four foraging bouts), the experiment started. A pre-test was conducted in which bees were presented during 30 s with both visual stimuli simultaneously to check for spontaneous preferences (Fig. 2a). The stimulus position (left or right arm) was randomized between bees (see above). One minute of darkness followed this pre-test and the differential conditioning started following the same procedure as at the treadmill.

Bees were subjected to 12 conditioning trials (6 CS+ and 6 CS− trials in a pseudorandom succession, which was the same for all trained bees and reproduced the one used at the treadmill, Fig. 2a). One stimulus (CS+, paired with 1 M sucrose, or CS−, paired with 60 mM quinine) was visible at a time in one arm of the maze while the other arm presented a black screen associated with an empty feeder. The side of stimulus presentation was varied between trials in the same way as in the treadmill. Bees were allowed to drink the sucrose solution *ad libitum* before leaving for the hive. Because of this, the time spent in the maze during CS+ trials was longer than for CS− trials. Yet, for the sake of comparative analyses, responses were quantified during the first 30 s in both types of trials. The bees were in full control of the interstimulus interval and the intertrial interval. The latter was defined by the return time of the bee to the maze and lasted usually 3 to 5 min.

After the last conditioning trial and upon the next return to the maze, bees were subjected to a post-test in which both stimuli, CS+ and CS−, were presented simultaneously during 30 s in the absence of reinforcement (Fig. 2a).

#### Data analysis and statistics

During the tests and conditioning trials, we quantified the bees’ first choice and calculated on this basis the percentage of bees belonging to the three categories defined for the treadmill experiments (individuals choosing the CS+, the CS− or not making a choice; see above). The first choice was defined as the first arm (CS+, CS−) entered by a walking bee upon arrival to the maze, which was followed by a touch of the screen displaying the stimulus at the end of the arm. The bees’ performance was represented with its corresponding 95% confidence interval. The time spent within each arm during the 30 s of stimulus presentation was also quantified and represented in terms of medians with corresponding quartiles, as it did not follow a normal distribution.

For the pre-test and the post-test, the proportions of individuals choosing the CS+, the CS− or not making a choice were compared within groups by means of a generalized linear mixed model (GLMM) for binomial family in which the individual identity (Bee) was considered as a random factor (individual effect). The Tukey method was used for multiple comparisons; z values with corresponding degrees of freedom are reported throughout for this kind of analysis. Comparisons between groups were done using a GLMM for binomial family with Group, Choice and interaction Group ^*^ Choice as main effects; χ^2^ values with corresponding degrees of freedom are reported throughout for these analyses. The time spent in the arms displaying the CS+ and the CS− and its variation between the pre-test and the post-test was analyzed by means of a Wilcoxon U test.

For the acquisition, the proportion of bees, which chose the CS+ or the CS−, was analyzed by means of a generalized linear mixed model (GLMM) for binomial family in which Trial was considered as a continuous factor (Trial effect) and the individual identity (Bee) as a random factor (individual effect). For within-group analyses, χ^2^ values are reported for CS effect, Trial effect and CS ^*^ Trial effect. Multiple comparisons were performed using Tukey’s method (z values reported).

All statistical analyses were done with R 3.2.3 (R Core Team 2016). Packages lme4 and lsmeans were used for GLMMs and Tukey’s method for multiple comparisons, respectively.

### Experiments on the treadmill

#### Preparation of bees for experiments on the treadmill

Captured bees were brought to the laboratory where they were placed on ice for five minutes in order to reduce their activity. This facilitated the fixation of a vertical tethering attachment to the thorax of each bee. The attachment was composed of a toothpick and a small piece of metal glued to the thorax with UV-curing opaque dentine (3 M Symphony D01-D05, DT&SHOP, Bad Bocklet, Germany) (Fig. 1b). The toothpick was used to clip the bee to a micromanipulator (M3301, WPI, Sarasota, USA) and adjust its position on the treadmill. Each bee was placed in a dark, humid box, and attached to the box’s lid by its tether for at least one hour before the start of the experiment, in order to habituate it to the tethering. These steps were done in a dark room under red light.

#### Treadmill

The apparatus (Fig. 1c,d) was composed of a polystyrene ball (diameter: 10 cm, weight: 8 g) held by a 3D-printed support and floating on a constant air flow produced by an air pump (air flow: 555 ml/s; Aqua Oxy CWS 2000, Oase, Wasquehal, France). To accurately position the bee on top of the ball, the tether was mounted on the micromanipulator. The treadmill was placed in front of a semi-cylindrical screen of tracing paper (height: 25 cm; distance to the bee: 7 cm), onto which visual stimuli were projected using the same video projector used for projecting visual stimuli in the mini maze. A set of mirrors was used to present stimuli on the lateral parts of the screen without deformation. The stimuli appeared on the screen at 50° to the left and right of the central axis of the bee body. To have full control of temporal parameters in our experiments (CS time, US time, trial duration and intertrial interval), we used an open-loop condition for both spatial variables, direction and distance, i.e., the bees’ movement modified neither the relative position, nor the apparent size of the stimuli.

The treadmill translates the movements of the walking bee into rotations of the ball. These were recorded by two infrared optic-mouse sensors (Logitech M500, 5700 dpi, Logitech, Lausanne, Switzerland), which were placed on the ball support, at 90° from each other. The rotational speed of the ball around the vertical axis was calculated to account for the directional walking movements of the bee (“instant heading”; one data point every 250 ms) using the following equation:

$$Instant\,heading=-(\frac{\frac{X1+X2}{2}}{5700})\,\ast \,(\frac{25.4\,\ast \,180}{R\pi })$$

X1 and X2 are, respectively, the translational movement in dots recorded in the horizontal axis of each sensor and 5700 is the sampling rate of the sensors in dots/inch. Multiplying the obtained value by 25.4 allows conversion into millimeters while multiplying it by 2πR (with R being the radius of the ball) converts the measured distance from millimeters into radians. Finally, multiplying by 180/π converts radians to degrees.

A *cumulative heading* was calculated for the whole duration of each trial or test session by summing up the instant headings. This measure was used to categorize the walking behavior of bees as ‘right choice’ if the cumulative heading was larger than 50°, ‘left choice’ if it was smaller than −50°, or ‘no choice’ if it was between −50° and 50°. The cutoff value of 50° corresponds to the position of the stimuli on the screen with respect to the bee’s central axis (see above) when the insect is placed on the tracking ball. This value corresponds consequently to the rotation that a bee should make to orient its body axis towards the stimulus. Cumulative headings could exceed 360° during a test/trial and were thus represented as multiples of a 360° rotation.

#### Visual stimuli

The visual stimuli to be discriminated were a blue square (3 × 3 cm; RGB:-1,-1, 1, with dominant wavelength at 450 nm) and a green disc (diameter: 3.4 cm; RGB:-1, 0.2,-1, with dominant wavelength at 530 nm) (Fig. 2b). Stimulus intensity was measured at the level of the bee eye, inside the arena. It was the same as in the mini maze experiments, i.e. 14000 µW/cm^2^ for the blue square and 1800 µW/cm^2^ for the green disc. These values were chosen to suppress a homogeneous original attraction towards the green light. This was possible in all experimental groups, but it induced in 3 out of 8 cases a higher proportion of bees preferring the blue square to the green disc (*paired group* in Fig. 4 and *distilled-water* and *quinine group* in Fig. 7). In the other 5 cases (*freely-walking bees* in Fig. 3, *CS* and *US groups* in Fig. 4, and *NaCl* and *dry-toothpick groups* in Fig. 7) the proportion of bees preferring the blue square and the green disc did not differ. Preliminary experiments showed that higher-intensity values for the green disc reestablished a homogeneous preference for this stimulus, and were, therefore, discarded.

Stimuli were equal in their total area (9 cm^2^) and subtended a similar visual angle to the bee eyes (square, from edge to edge: 24.2°; disc: 27.3°). As in the mini maze experiment, this angular range ensures that bees engaged their chromatic vision to achieve the visual discrimination^50,51^. Both during the training and during the pre- and the post-tests, the visual stimuli were presented during 30 s.

#### Conditioning and testing at the treadmill

Experiments (Fig. 2b) started with a ‘pre-test’ in which the stimuli to be discriminated were presented simultaneously during 30 seconds (at 50° on each side of the bee’s body axis) to check for spontaneous preferences. The stimulus position (left or right of the bees’ body axis) was randomized between bees. After this pre-test, the bees were offered one minute without stimulation (black screen). Thereafter, the conditioning protocol started.

Bees were trained to discriminate the two visual stimuli based on their different reinforcement outcomes (differential conditioning). They experienced a succession of 12 trials in which they were presented either with one stimulus (CS+) paired with 1 M sucrose solution or with the other stimulus (CS−) paired with 60 mM quinine solution. In each trial, the CS was displayed during 30 s either at −50° or at +50° from the bee’s body axis. The stimulus side was pseudorandomized from trial to trial and the side sequence was the same from bee to bee (L, L, R, R, L, L, L, R, R, L, R, R). The US (sucrose or quinine) was provided during the last 5 s of CS presentation by means of a wooden toothpick that contacted the antenna on the side of the visual stimulus. The US was then delivered to the proboscis when it was extended. If no extension occurred upon delivery of quinine (or other negative reinforcement, see below), the solution was nevertheless approached to the proboscis in the first two trials; afterwards, negative reinforcements remained at the level of the antenna to avoid excessive disturbance. Delivery of reinforcements via a toothpick could provide uncontrolled visual cues. However, this delivery procedure was used for both appetitive and aversive reinforcements and did not offer discriminative cues. Moreover, acquisition data reported correspond to conditioned responses to the visual stimuli occurring *before* reinforcement delivery, i.e. before toothpick use. In addition, during the tests, no toothpick was presented. These facts exclude the potential confounding factor of toothpick movement in the performances reported.

The bees experienced 6 CS+ and 6 CS− trials in a pseudorandom sequence (CS+, CS−, CS+, CS−, CS−, CS+, CS−, CS−, CS+, CS+, CS−, CS+), which was the same from bee to bee. Trials were separated by an intertrial interval of one minute. Such a short interval was chosen to diminish the impact of a possible decrease in the bee’s motivation to walk. If a bee did not respond with a proboscis extension to any sucrose-rewarding trial, or if it did not walk during the experiment, it was discarded from the analyses (<10% in each group).

One minute after the end of the last conditioning trial, bees were subjected to a ‘post-test’ in which they saw simultaneously the CS+ and the CS− during 30 seconds in the absence of reinforcement. Performance in the post-test revealed if bees learned the discrimination or not, as it could be compared with performance in the pre-test before conditioning.

#### Experiment 2: visual learning at the treadmill

Bees were randomly allocated to three different groups (Fig. 2c): (1) the *paired group* experienced a differential conditioning protocol, which followed the steps described above; two subgroups were trained in order to balance reinforcement experience with the green disc and the blue square (i.e., one subgroup experienced the green disc rewarded and the blue square punished, while the other subgroup experienced the reversed contingency); (2) the *CS group* experienced only visual stimulation matching that of the *paired group*, i.e. 12 CS presentations (6 green discs and 6 blue squares) but without any US; (3) the *US group* experienced a sequence of 12 US presentations (6 sucrose and 6 quinine presentations) matching that of the *paired group*, i.e. a given US was delivered on the right or left antenna, corresponding to a theoretical CS stimulation on the right or on the left of the screen, respectively.

#### Experiment 3: the effect of negative US on visual learning at the treadmill

Bees were randomly allocated to four groups that experienced the conditioning schedule of the *paired group* of Experiment 1. All groups received 1 M sucrose as positive US but differed in the nature of the negative US associated with the CS−, which could be a solution of 60 mM quinine, 3 M NaCl, distilled water or contact with a dry toothpick. The *quinine group* therefore reproduces the treatment experienced by the *paired group* of Experiment 1. All groups were balanced in terms of the association stimulus (green disc, blue square) - reinforcement (sucrose, negative reinforcement).

#### Data analysis and statistics

During the tests and conditioning trials, the response of the bee was categorized as ‘choice of the right side’, ‘choice of the left side’ or ‘absence of choice’, depending on the value of the cumulative heading (see above). In the *paired groups*, the bee’s choice was then recorded as correct (CS+ side), incorrect (CS− side) or as a non-choice. In the *CS group*, it was recorded as CS1 choice, CS2 choice or non-choice. In the US group, it was recorded as ‘theoretical CS+ ’ choice, ‘theoretical CS−’ choice or non-choice. Within each category, individual data were converted into a binomial format (0 or 1 if the bee’s choice belonged to this category or not, respectively) to calculate the proportion of individuals which chose the CS+, the CS−, or which didn’t make a choice. Data were bootstrapped^49^ to plot these proportions ± their corresponding 95% confidence interval. Analyses were also performed on the cumulative heading itself, quantified during the 30 s of stimulus presentation. In this case, data did not follow a normal distribution. They are thus presented as medians with corresponding quartiles.

For the pre-test and the post-test, the proportions of individuals choosing first the CS+, the CS− or not making a choice during the 30 s of stimulus presentation were computed. This duration was chosen after determining that shorter durations did not induce significant differences between these categories. Indeed, although some bees chose stimuli during the first seconds of stimulus presentations, their number was not sufficient to generate significant differences. This is well illustrated by a reanalysis of the *quinine* and the *NaCl groups* of Fig. 7 using only the first 10 s of stimulus presentation (Supplementary Fig. S7). These two groups are interesting as the former exhibited a learning-induced increase in the proportion of CS+ choosers with respect to CS− choosers (Fig. S7b), while the latter did not show differences in the proportions of CS+ choosers, CS− choosers and non-choosers after conditioning (Fig. S7h). After reducing the period of analysis to the first 10 s, significance was lost in the *quinine group* (Fig. S7d) while the results of the *NaCl group* remained non-significant (Fig. S7j). Restricting the analysis to the subsequent period of 10–30 s did not change these results. The *quinine group* did not reach the clear significant difference visible when considering the entire 30 s period (Fig. S7f), and the *NaCl group* maintained its non-significant distribution (Fig. S7l). These results illustrate why the 30 s period was appropriate for computing preferences based on a cumulative proportion of bees choosing either stimulus, or not making a choice.

Test proportions were compared within groups by means of a generalized linear mixed model (GLMM) for binomial family in which the individual identity (Bee) was considered as a random factor (individual effect). The Tukey method was used for multiple comparisons; z values with corresponding degrees of freedom are reported throughout for this kind of analysis. Comparisons between groups were done using a GLMM for binomial family with Group, Choice and interaction Group ^*^ Choice as main effects; χ^2^ values with corresponding degrees of freedom are reported throughout for these analyses. The cumulative heading was compared with a theoretical orientation of 0° (no choice) by means of a one-sample Mann Whitney test. Furthermore, the within-group variation in cumulative heading between the pre-test and the post-test was analyzed by means of a Wilcoxon U rank test. Cumulative-heading performances were compared between groups using a Kruskal-Wallis test.

For the acquisition, the proportion of bees which chose the CS+ or the CS− was analyzed by means of a generalized linear mixed model (GLMM) for binomial family in which Trial was considered as a continuous factor (Trial effect) and the individual identity (Bee) as a random factor (individual effect). For within-group analyses, χ^2^ values are reported for CS effect, Trial effect and CS ^*^ Trial effect. Multiple comparisons were performed using Tukey’s method (z values reported).

All statistical analyses were done with R 3.2.3 (R Core Team 2016). Packages lme4 and lsmeans were used for GLMMs and Tukey’s method for multiple comparisons, respectively.

## Electronic supplementary material

Supplementary Information
