# Supplementary material for: Associative visual learning by tethered bees in a controlled visual environment
Source: Sci Rep. 2017 Oct 10;7:12903. doi: 10.1038/s41598-017-12631-w (PMC5635106; doi:10.1038/s41598-017-12631-w)
Supplement: Supplementary file 1 — Supplementary Information [file 41598_2017_12631_MOESM1_ESM.pdf]

Supplementary Information

Associative visual learning by tethered bees in a controlled visual environment

Alexis Buatois[1], Cécile Pichot[1], Patrick Schultheiss[1], Jean-Christophe Sandoz[2], Claudio R. Lazzari[3], Lars Chittka[4], Aurore Avarguès-Weber[1,]* & Martin Giurfa[1,]*

*senior authorship shared

[1]: Research Centre on Animal Cognition, Center for Integrative Biology, CNRS, University of Toulouse, 118 route de Narbonne, F-31062 Toulouse cedex 09, France

[2]: Laboratory Evolution Genomes Behavior and Ecology, CNRS, Univ Paris-Sud, IRD, University Paris Saclay, F-91198, Gif-sur-Yvette, France

[3]: Institut de Recherche sur la Biologie de l'Insecte, UMR 7261 CNRS, University François Rabelais of Tours, F-37200 Tours, France

[4]: Queen Mary University of London, School of Biological and Chemical Sciences, Biological and Experimental Psychology, Mile End Road, London E1 4NS, United Kingdom

Figure S1

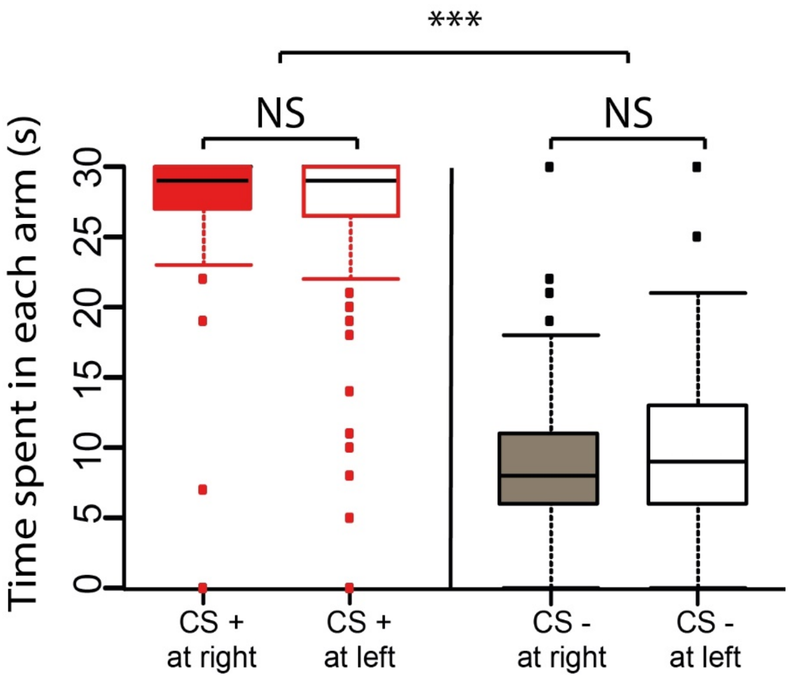

Figure S1. Experiment 1 (miniature maze). Time spent in each arm of the miniature maze (in seconds; median, quartiles and extreme values) during the 30 s of CS+ or CS- presentation in the 12 conditioning trials (6 for each alternative). For each stimulus type, there were three presentations on the right and three presentations on the left side of the screen. The graphs show the response of the bees (n = 21) to each stimulus alternative when it was on the right (pooled data of the 3 presentations) vs. when it was on the left (pooled data of the 3 presentations). Red boxplot, full: CS+ at right; red boxplot, empty: CS+ at left. Dark-grey boxplot, full: CS- at right; dark-grey boxplot, empty: CS- at left. In all four cases, the time spent in each arm was significantly different from zero (one-sample Mann-Whitney test; $CS+_{right}$: $U= 1891$, $p < 0.0001$; $CS+_{left}$: $U= 1891$, $p < 0.001$; $CS-_{right}$: $U= 1830$, $p < 0.0001$; $CS-_{left}$: $U= 1891$, $p < 0.0001$). Bees spent the same time in the left and right arms when they saw the CS+ (Wilcoxon rank test; $U =392$, $p= 0.11$) and the CS- ($U= 681$, $p= 0.11$). Nevertheless, this time was significantly longer in the case of the CS+ (Wilcoxon rank test; $U= 148$, $p< 0.0001$). Indeed, bees spent most of the CS+ stimulation time (30 s) in the CS+ arm and did practically not visit the empty arm. On the contrary, they spent only between 5 and 10 s in the CS- arm during the CS- stimulation.

Figure S2

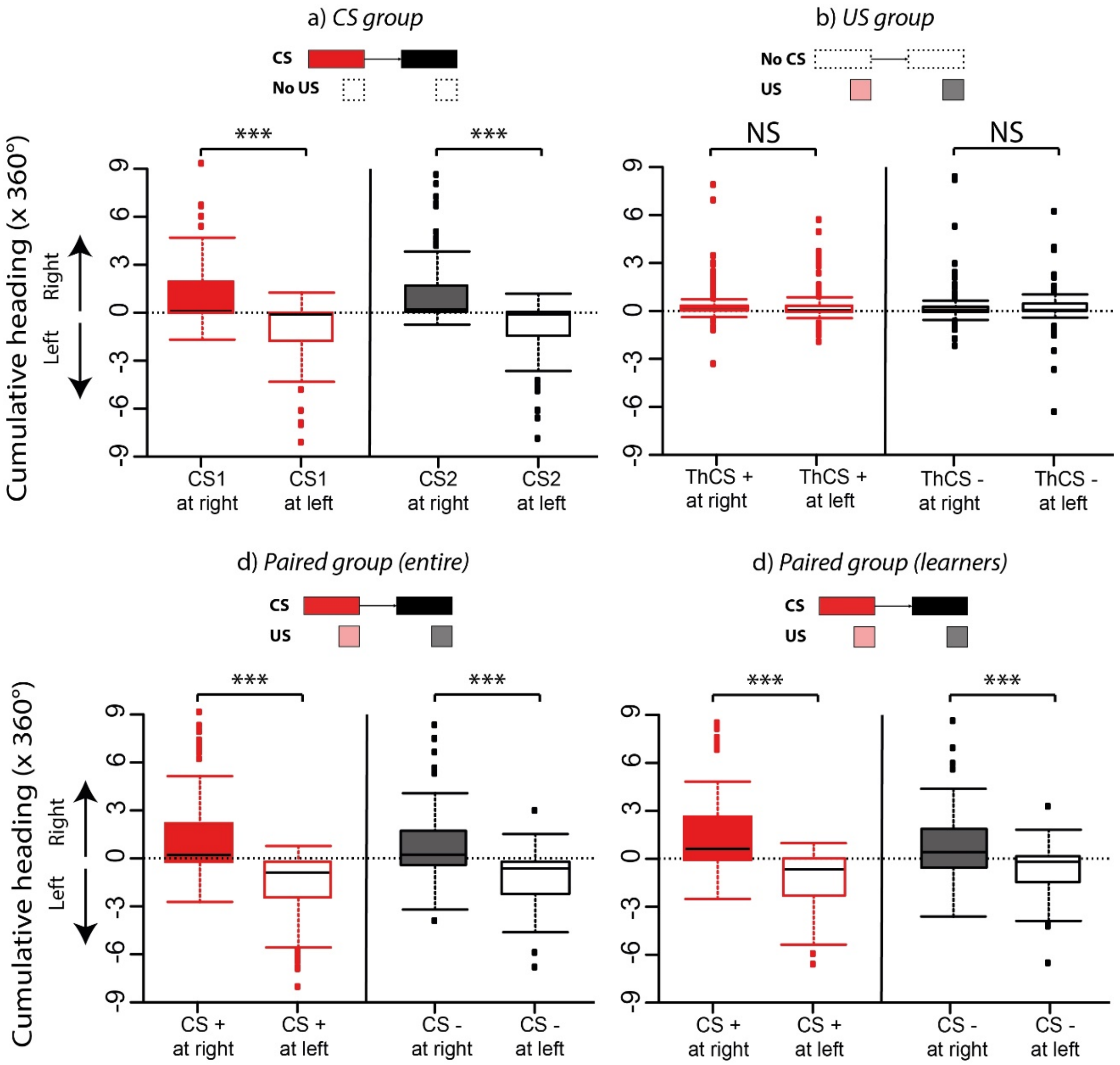

Figure S2. Experiment 2 (spherical treadmill). Cumulative heading (in degrees, median, quartiles and extreme values) for the two stimuli offered to the bees during the 12 conditioning trials (6 for each alternative). The values reported correspond to multiples of a 360° rotation. They were quantified during the 30 s of stimulus presentation. For each stimulus type (e.g. CS+ and CS- in the *paired group*), there were 3 presentations on the right and three presentations on the left side of the screen. The graphs show the response of the bees to each stimulus alternative when it was on the right (pooled data of the 3 presentations; median and quartiles) vs. when it was on the left (pooled data of the 3 presentations; median and quartiles). **(a)** *CS group* (n = 32). For these bees, there were only visual stimulations (CS1, CS2) without US. Red boxplot, full: CS1 at right; red boxplot, empty: CS1 at left. Dark-grey boxplot, full: CS2 at right; dark-grey boxplot, empty: CS2 at left. Bees of the *CS group* always oriented towards the stimulus that was visible on the screen, be it CS1 or CS2. In all cases, the cumulative heading was significantly different from a theoretical orientation of 0° (one-sample Mann-Whitney test; $CS1_{right}$: U

= 3834, $p < 0.0001$; $CS1_{left}$: U = 691, $p < 0.001$; $CS2_{right}$: U = 3883, $p < 0.0001$; $CS2_{left}$: U = 809, $p < 0.001$). The change in heading depending on stimulus side was significant for the CS1 (Wilcoxon rank test; U = 4137, $p < 0.0001$) and for the CS2 (U = 4041, $p < 0.0001$). In other words, when bees saw a stimulus on the right side of the screen, they headed towards the right and when they saw it on the left side they headed towards the left. **(b)** *US group* (n = 32). Bees in this group maintained a rather straightforward trajectory irrespectively of the US received just after performance recording (sucrose or quinine solution). The period before sucrose delivery is termed 'Theoretical CS+' (ThCS+) and that before quinine delivery is termed 'Theoretical CS-' (ThCS-). The cumulative heading was significantly different from a theoretical orientation of 0° only for the $ThCS+_{right}$, i.e. prior to sucrose delivery on the right antenna (U = 3017, p = 0.01) and for the $ThCS-_{left}$, i.e. prior to quinine delivery on the left antenna (U = 2954, p = 0.023). The other two situations, $ThCS+_{left}$ and $ThCS-_{right}$, i.e. prior to sucrose delivery on the left antenna, and prior to quinine delivery on the right antenna, resulted in headings that did not differ from 0° ($ThCS+_{left}$: U = 2670, p = 0.21; $ThCS-_{right}$: U = 2586, p = 0.35). The cumulative heading did not differ between situations ($ThCS+_{right}$ vs. $ThCS+_{left}$: U = 2748, p = 0.12; $ThCS-_{right}$ vs. $ThCS-_{left}$: U = 2286, p = 0.88). **(c)** Entire *Paired group* (n = 38). Red boxplot, full: CS+ at right; red boxplot, empty: CS+ at left. Dark-grey boxplot, full: CS- at right; dark-grey boxplot, empty: CS- at left. Bees of the paired group always oriented towards the stimulus that was visible on the screen, be it CS+ or CS-. Thus, in all four cases, the cumulative heading was significantly different from a theoretical orientation of 0° (one-sample Mann-Whitney test; $CS+_{right}$: U= 5367, $p < 0.0001$; $CS+_{left}$: U= 844, $p < 0.001$; $CS-_{right}$: U= 4743, $p < 0.0001$; $CS-_{left}$: U= 1138, $p < 0.0001$). Moreover, the change in heading depending on the side of presentation was significant both for the CS+ (Wilcoxon rank test; U =5797, $p < 0.0001$) and for the CS- (U = 5425, $p < 0.0001$). In other words, when a stimulus was displayed on the right side of the screen, bees headed towards the right, and when it was shown on the left side, they headed towards the left. **d)** Learners of the *paired group* (n = 22). Red boxplot, full: CS+ at right; red boxplot, empty: CS+ at left. Dark-grey boxplot, full: CS- at right; dark-grey boxplot, empty: CS- at left. These bees always oriented towards the stimulus that was visible on the screen, be it CS+ or CS-. In all four cases, the cumulative heading was significantly different from a theoretical orientation of 0° (one-sample Mann-Whitney test; $CS+_{right}$: U= 1758, $p < 0.0001$; $CS+_{left}$: U= 349, $p < 0.001$; $CS-_{right}$: U= 1455, $p < 0.03$; $CS-_{left}$: U= 523, $p < 0.001$). The change in heading depending on the side of presentation was significant both for the CS+ (Wilcoxon rank test; U = 1878, $p < 0.0001$) and for the CS- (U = 1707, $p < 0.0001$). Thus, learners behaved like the entire *paired group*: they simply headed towards the visible stimulus be it on the right or on the left. *** $p < 0.0001$; NS: non-significant

Figure S3

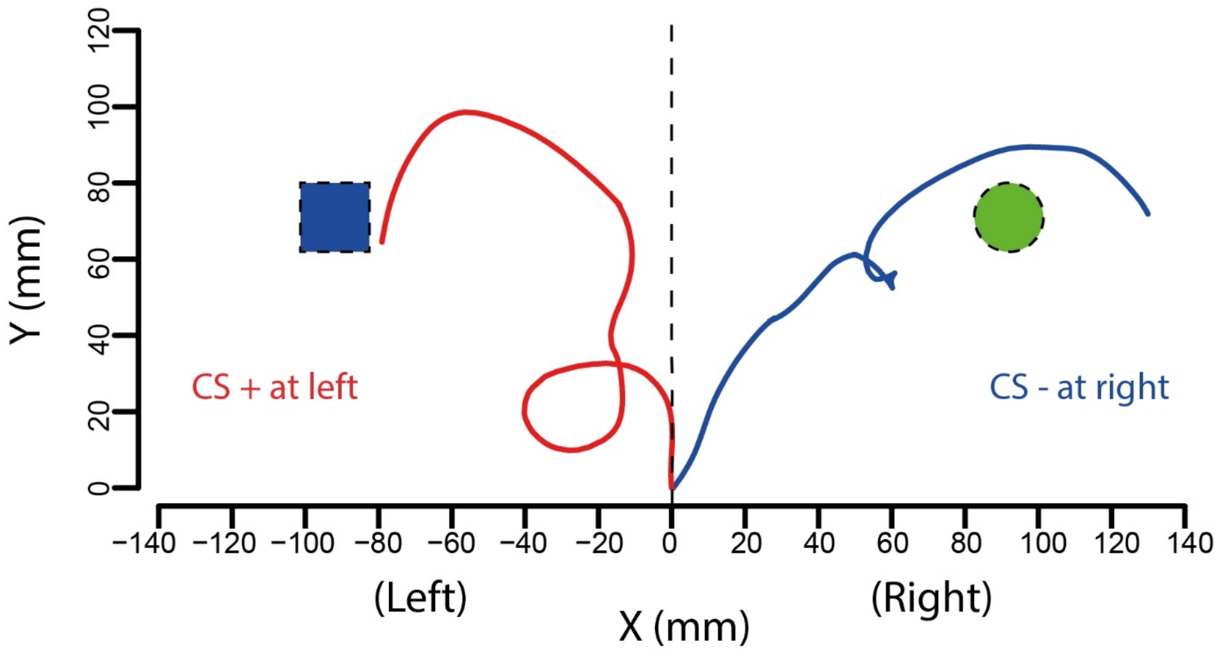

Figure S3. Experiment 2 (spherical treadmill). Example of two trajectories of a bee in the *paired group*, which correspond to two conditioning trials, one in which the CS+ was shown at the left (red trajectory), and another in which the CS- was shown at the right (blue trajectory). The dashed square and disc on the left and right, respectively, indicate the fictive position of the stimuli (one shown at a time during training). In both cases, the bee headed towards the visual stimulus presented, irrespectively of the US associated with the visual stimulus, thus showing either a phototactic effect or an object-fixation effect. Trajectories were calculated following Seelig et al.[1].

For the walking displacements on the X-axis (dX; right – left), we used the following equation:

$$dX = (Y1 + Y2) * \cos(45°) * sin\ (heading) - (Y1 - Y2) * \sin(45°) * \cos(heading)$$

For the displacements on the Y-axis (dY; forward), we used the following equation:

$$dY = -(Y1 + Y2) * \cos(45°) * cos\ (heading) - (Y1 - Y2) * \sin(45°) * \sin(heading)$$

with Y1 and Y2 being the vertical movements detected by the sensors 1 and 2, respectively. See the main text to see how the bee’s heading was calculated.

Figure S4

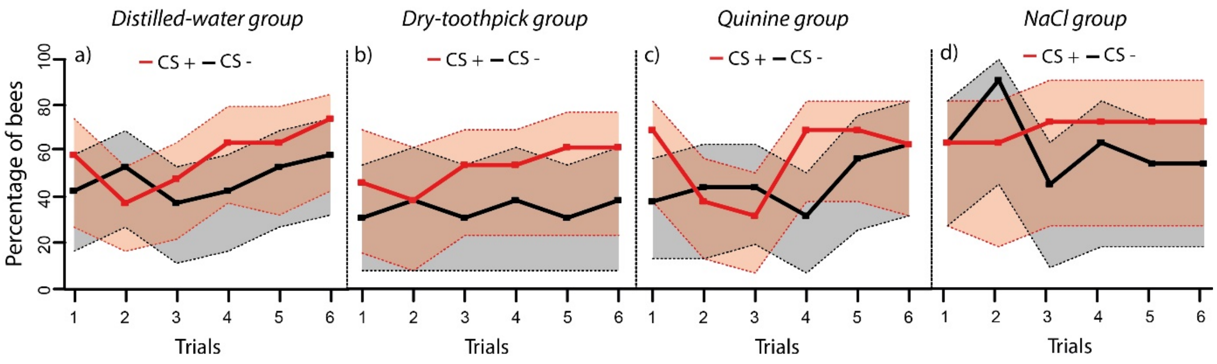

Figure S4. Experiment 3 (spherical treadmill). Acquisition performance (percentage of learners choosing the CS+, red curve, and the CS-, black curve) during the 12 conditioning trials (6 for each CS alternative). **(a)** Learners of the *distilled-water group* (n=19). **(b)** Learners of the *dry-toothpick group* (n=13). **(c)** Learners of the *quinine group* (n=16). **(d)** Learners of the *NaCl group* (n=11). In all four groups, the 95% confidence interval is shown (dashed lines; in pink for the CS+ curve and in grey for the CS- curve). Learners in all groups exhibited a strong tendency to choose the single visual stimulus presented, be it CS+ or CS-, from the very first trial (Figs. 8 a, b, c, d) and kept doing so during the 12 trials. Consequently, the bees' performance did not show any variation along trials (trial effect; *distilled water group*: $\chi^2 = 3.57$, df: 5, p = 0.61; *dry-toothpick group*: $\chi^2 = 5.75$, df: 5, p = 0.33; *quinine group*: $\chi^2 = 7.40$, df: 5, p = 0.19; *NaCl group*: $\chi^2 = 3.88$, df: 5, p = 0.57). For the same reason, the learning curves did not show any significant evidence of CS discrimination (CS effect; *distilled water group*: $\chi^2 = 0.09$, df: 1, p = 0.77; *dry-toothpick group*: $\chi^2 = 0.36$, df: 1, p = 0.55; *quinine group*: $\chi^2 = 0.81$, df: 1, p = 0.37; *NaCl group*: $\chi^2 = 0.38$, df: 1, p = 0.54). In all four groups, the interaction CS x trial was also not significant (*distilled water group*: $\chi^2 = 4.21$, df: 5, p = 0.52; *dry-toothpick group*: $\chi^2 = 4.40$, df: 5, p = 0.49; *quinine group*: $\chi^2 = 4.75$, df: 5, p = 0.45; *NaCl group*: $\chi^2 = 4.43$, df: 5, p = 0.49). This indicates that in all groups there was an apparent absence of learning and that the responses to both CS types followed the same dynamics. A global analysis including all four groups confirmed that performance was homogeneous irrespective of the US associated with the CS- (group effect; df: 3, $\chi^2 = 2.65$, p = 0.44; group x trial x CS effect; df: 35, $\chi^2 = 24.7$, p = 0.91). These results can be explained based on positive phototaxis or on an object-fixation response, which drove the bees towards the single visual stimulus displayed on the screen during a training trial.

Figure S5

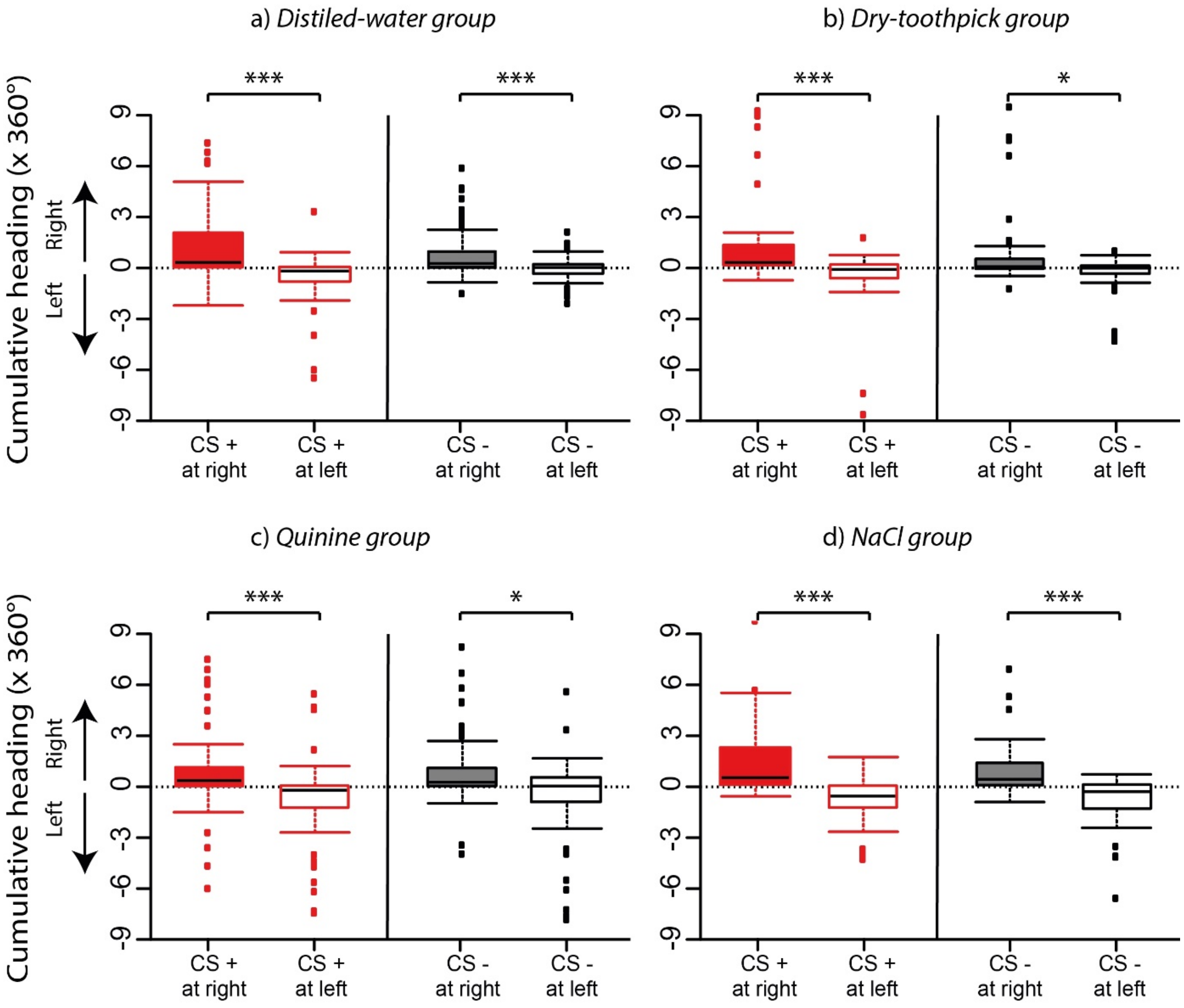

Figure S5. Experiment 3 (spherical treadmill). Cumulative heading (in degrees, median, quartiles and extreme values) for the CS+ and the CS- offered to the bees during the 12 conditioning trials (6 for each alternative). The values reported correspond to multiples of a 360° rotation. They were quantified during the 30 s of stimulus presentation. For each CS type, there were three presentations on the right and three presentations on the left side of the screen. The graphs show the response of learner bees to each CS when it was on the right (pooled data of the 3 presentations; median and quartiles) vs. when it was on the left (pooled data of the 3 presentations; median and quartiles). Red boxplot, full: CS+ at right; red boxplot, empty: CS+ at left. Dark-grey boxplot, full: CS- at right; dark-grey boxplot, empty: CS- at left. **(a)** Learners of the *distilled-water group* (n = 19). Bees of this group headed towards the right when a visual target was shown on the right of the screen and towards the left when it was shown on the left of the screen, irrespectively of the CS type. Thus, in three of four possible situations, the cumulative heading was significantly different from a theoretical orientation of 0° (one-sample Mann-Whitney test; $CS+_{right}$: $U = 1296$, $p < 0.0001$; $CS+_{left}$: $U = 286$, $p < 0.0001$; $CS-_{right}$: $U = 1287$, $p < 0.001$; $CS-_{left}$: U =

681, $p = 0.25$). The change in heading depending on stimulus side was significant for the CS+ (Wilcoxon rank test; $U = 1344$, $p < 0.0001$) and for the CS- ($U = 1320$, $p < 0.0001$). **(b)** Learners of the *dry-toothpick group* (n=13). Bees of this group also headed towards the right when the CS+ was shown on the right of the screen and towards the left when it was shown on the left of the screen. No significant heading was found for the CS- ($CS+_{right}$: $U = 645$, $p < 0.001$; $CS+_{left}$: $U = 205$, $p < 0.01$; $CS-_{right}$: $U = 507$, $p = 0.10$; $CS-_{left}$: $U = 268$, $p = 0.09$). The change in heading depending on stimulus side was significant for the CS+ ($U = 646$, $p < 0.0001$) and for the CS- ($U = 583$, $p < 0.01$). **(c)** Learners of the *quinine group* (n = 16). Bees of this group exhibited a cumulative heading that was significantly different from a theoretical orientation of 0°in three of four possible situations ($CS+_{right}$: $U = 881$, $p < 0.001$; $CS+_{left}$: $U = 264$, $p < 0.001$; $CS-_{right}$: $U = 924$, $p < 0.001$; $CS-_{left}$: $U = 506$, $p = 0.41$). The change in heading depending on stimulus side was significant for the CS+ ($U = 993$, $p < 0.0001$) and for the CS- ($U = 857$, $p < 0.01$). **(e)** Learners of the *NaCl group* (n=11). Bees headed toward the CS regardless of its position and its associated US ($CS+_{right}$: $U = 506$, $p < 0.0001$; $CS+_{left}$: $U = 78$, $p < 0.0001$; $CS-_{right}$: $U = 494$, $p < 0.0001$; $CS-_{left}$: $U = 119$, $p < 0.01$). The change in heading depending on stimulus side was significant for the CS+ ($U = 525$, $p < 0.0001$) and for the CS- ($U = 520$, $p < 0.0001$). *** $p < 0.0001$; * $p < 0.01$; NS: non-significant.

Figure S6

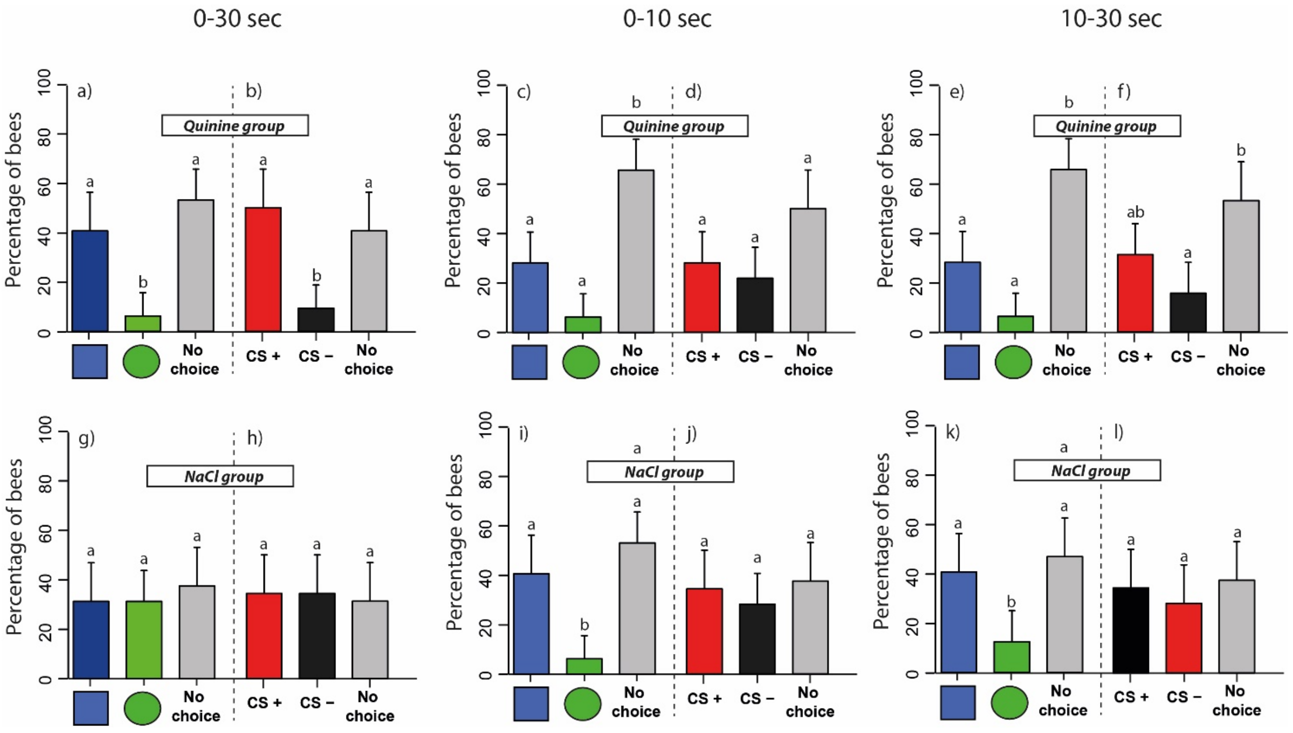

Figure S6. Pre- and post-test performances under shorter visual-stimulus periods (0-10 s, middle panels, and 10-30 s, right panels). **Left panels show performances computed using the entire stimulus period of 30 s.** Choice performance (percentage of bees choosing a given stimulus or not making a choice + 95% confidence interval) during the pre-test. **(a,b)** Pre- and post-tests performances of the *quinine group* computed over the entire 30 s of stimulus duration (as in Fig. 7, main text). **(g,h)** Same for the *NaCl group* (as in Fig. 7, main text). **(c,d)** Pre- and post-tests performances of the *quinine group* computed over the first 10 s of stimulus duration. Pre-test: blue square vs. green disc: z_{1161} = -2.13, p = 0.08, blue square vs. no choice: z_{1161} = -2.93, p = 0.009, green disc vs. no choice: z_{1161} = -4.09, p = 0.0001. Post-test: CS+ vs. CS-: z_{1161} = -0.58, p = 0.83, CS+ vs. no choice: z_{1161} = -1.77, p = 0.18, CS- vs. no choice: z_{1161} = -2.29, p = 0.06. The use of a shorter period for computing the bee choices did not result in significant differences between choice categories **(i,j)** Pre- and post-tests performances of the *NaCl group* computed over the first 10 s stimulus duration. Pre-test: blue square vs. green disc: z_{1161} = -2.86, p = 0.01, blue square vs. no choice: z_{1161} = -0.99, p = 0.58, green disc vs. no choice: z_{1161} = -3.49, p = 0.001. Post-test: CS+ vs. CS-: z_{1161} = -0.53, p = 0.85, CS+ vs. no choice: z_{1161} = -0.26, p = 0.96, CS- vs. no choice: z_{1161} = -0.79, p = 0.70. As for the *quinine group*, the use of a shorter stimulus duration did not result in significant differences between choice categories in the post-test. **(e,f)** Pre- and post-tests performances of the *quinine group* computed over the subsequent 10 - 30 s stimulus duration. Pre-test: blue square vs. green disc: z_{1161} = -2.13, p = 0.08, blue square vs. no choice: z_{1161} = -2.93, p = 0.01, green disc vs. no choice: z_{1161} = -4.09, p = 0.0001. Post-test: CS+ vs. CS-: z_{1161} = -1.45, p = 0.31, CS+

vs. no choice: z_{1161} = -1.75, p = 0.18, CS- vs. no choice: z_{1161} = -3.01, p = 0.007. No clear significant difference between choice categories was visible in the post-test when restricting the analysis to this period. **(k,l)** Pre- and post-tests performances of the *NaCl group* computed over the subsequent 10 - 30 s stimulus duration. Pre-test: blue square vs. green disc: z_{1161} = -2.43, p = 0.04, blue square vs. no choice: z_{1161} = -0.5, p = 0.87, green disc vs. no choice: z_{1161} = -2.84, p = 0.013. Post-test: CS+ vs. CS-: z_{1161} = -0.54, p = 0.85, CS+ vs. no choice: z_{1161} = -0.26, p = 0.96, CS- vs. no choice: z_{1161} = -0.79, p = 0.7. Again, no significant difference between the proportions of bees choosing the CS+ or the CS- was visible during the Post-test when restricting the analysis to this stimulus period. These results illustrate well why the 30 s period was appropriate for computing preferences based on a cumulative proportion of bees choosing either stimulus or not performing any choice.

References

1 Seelig, J. D. *et al.* Two-photon calcium imaging from head-fixed *Drosophila* during optomotor walking behavior. *Nat Meth* **8**, 184-184, doi:10.1038/nmeth0211-184b (2011).
